# Small siphophage binding to an open state of the LptDE outer membrane lipopolysaccharide translocon

**DOI:** 10.1073/pnas.2516650122

**Published:** 2025-11-26

**Authors:** Emily Dunbar, Robert Clark, Arnaud Baslé, Shenaz Allyjaun, Hector Newman, Julia Hubbard, Syma Khalid, Bert van den Berg

**Affiliations:** ^a^Biosciences Institute, The Medical School, Newcastle University, Newcastle upon Tyne NE2 4HH, United Kingdom; ^b^Department of Biochemistry, University of Oxford, Oxford OX1 3QU, United Kingdom; ^c^BicycleTx Limited, Cambridge CB21 6GS, United Kingdom

**Keywords:** bacteriology, structural biology, phage biology, cryo-EM, outer membrane

## Abstract

Bacteriophages are bacterial viruses that provide alternatives to small-molecule drugs to combat infections by antibiotic-resistant bacteria. To infect a bacterial host, a phage needs to bind to the bacterial surface via receptor binding proteins (RBPs), which are critical for determining host specificity. For functionally important receptors, the RBP–receptor interaction could be exploited via phage steering, where emerging bacterial resistance due to receptor modification could make bacteria less fit or virulent. Despite this, relatively little is known about RBP–receptor interactions. Here, we build on the recent discovery of coliphages that have the outer membrane (OM) lipopolysaccharide translocon LptDE as their terminal receptor and show via cryogenic electron microscopy that, surprisingly, the RBP of the small siphophage Oekolampad binds to a hitherto unobserved, open state of LptDE. The open lateral gate of LptD is occupied by a β-strand peptide originating from the degraded N-terminal jellyroll domain of LptD, suggesting the possibility of LptD inhibition via peptidomimetics. A structure of LptDE in complex with the superinfection exclusion (SE) protein Rtp45 of the Oekolampad-related phage Rtp shows a mechanism of SE where Rtp45-induced conformational changes in LptD resulting from steric clashes preclude RBP binding. Finally, analysis of spontaneous Oekolampad-resistant *Escherichia coli* mutants identifies mutations in LptD that abolish the LptDE–RBP interaction in vitro. SDS-EDTA sensitivity assays of the mutants show no major OM defects, consistent with largely preserved LptDE function, and suggesting that phage steering via LptDE might be challenging.

## Significance

The outer membrane (OM) of Gram-negative bacteria is a protective barrier generated by lipopolysaccharide (LPS), a complex glycolipid that makes up the outer leaflet of the OM. Following its synthesis and transport from the inner membrane, LPS is inserted into the OM by LptDE, an OM protein complex essential for most Gram-negative bacteria. LPS insertion requires a hitherto unobserved, open state of LptDE. Here, we report the unexpected finding that the receptor binding protein (RBP) of a bacteriophage binds to an open state of LptDE, allowing isolation and visualization via cryogenic electron microscopy. The lateral gate of the LptDE–RBP complex is occupied by a β-strand peptide, suggesting that the LptDE of pathogenic bacteria could be inhibited by peptidomimetics.

The increased threat of bacterial multidrug resistance has resulted in a renewed interest in phage therapy to combat pathogenic bacteria, especially difficult-to-treat Gram-negatives such as *Pseudomonas aeruginosa* and **Acinetobacter* baumannii* (1). For a bacteriophage to inject its genome and infect a cell, phage attachment (adsorption) to the host cell must take place. For most phages, this step requires phage receptor binding proteins (RBPs) located in the phage tail, which recognize and bind to specific bacterial surface molecules such as outer membrane proteins (OMPs), polysaccharides, and lipopolysaccharides (2). Initial reversible binding often precedes an irreversible binding step to a different, terminal receptor that results in phage DNA injection. Despite its importance for determining host specificity, relatively little is known about the molecular details of bacterial receptor recognition by phage RBPs, especially in the case of the abundant Siphophages, which have long and flexible, noncontractile tails and RBPs that often target OMPs. An exception is bacteriophage T5, which targets the *Escherichia coli* FhuA ferrichrome OM TonB-dependent transporter (TBDT) as its terminal receptor via its RBP pb5, and which employs the small periplasmic lipoprotein Llp for superinfection exclusion (SE) (3–5)

As with conventional antibiotic treatment of bacterial infections, phage therapy inevitably leads to bacterial resistance. In the example of phage T5 above, resistance could arise via, for example, shutting down FhuA expression by *E. coli*. This would likely be tolerated well, given that *E. coli* has six TBDTs for the acquisition of different iron–siderophore complexes (6), and under most conditions, the loss of one or several of those transporters would not be expected to greatly affect fitness. This latter point is a key aspect of phage steering, a strategy where phage therapy relies on the generation of resistance that renders the surviving bacteria either less virulent, less fit, or both (7–9). In the case of FhuA and phage T5, such a scenario is unlikely but might occur if ferrichrome were the most abundant source of iron available to *E. coli* at an infection site. However, for obvious reasons, phage steering is likely to be the most efficient for phages that target essential OMPs. The expression of such proteins cannot be shut down, and the generation of resistance might result in impaired function of the essential OMP and lower fitness or virulence. Only two OMPs are known to be essential in all or most Gram-negative bacteria: the BamA component of the β-barrel assembly machinery (BAM) (10–12) and the lipopolysaccharide translocon LptDE (13, 14). The latter is the destination of lipopolysaccharide (LPS) molecules that traverse from the inner membrane to the OM via the seven-membered Lpt (lipopolysaccharide transport) system (15). This consists of the LptBCFG ABC transporter that extracts the LPS molecule from the IM and hands it off to the periplasmic LptA protein (16, 17). Several copies of LptA link LptBCFG to LptDE in the OM, forming a protein bridge spanning the periplasmic space that functions as an ATP hydrolysis-driven conveyor belt for LPS molecules (18, 19). Once they arrive at the LptDE complex, the LPS molecules are inserted into the outer leaflet of the OM (20, 21) via a process for which the details are not clear, but which must involve a separation of the first (S1) and last (S26) β-strands of the LptD barrel at the front of the complex to allow the translocating LPS molecule to move laterally into the OM from the lumen of the LptDE complex (22, 23). So far, in contrast to BamA (24–27), there is no structural information available for an open state of LptDE. Moreover, phages targeting LptDE were only identified recently, when a library of 68 newly isolated coliphages (named the BASEL collection) was characterized together with several model phages and phage OMP receptors were identified via a panel of single OMP deletion strains (28). This approach failed for several small siphophages, but their receptor was subsequently identified as LptD via whole-genome sequencing of mutant strains that developed spontaneous resistance to those phages (28). The identification of LptD-targeting phages provides opportunities to investigate whether an essential OMP could potentially be exploited toward phage steering.

Here, we report the generation and cryo-EM structure determination of a complex between **Shigella* flexneri* LptDE (SfLptDE; >99% sequence identity with *E. coli* LptDE) and the RBP of the BASEL collection phage Oekolampad (bas018), which has LptD as terminal receptor (28). Oekolampad RBP (RBP_oeko_) binds to the extracellular face of an LptDE complex in which the LptD barrel has dramatically expanded at the front to form a wide channel connecting the periplasmic space to the extracellular environment. Moreover, the LptD lateral gate is occupied by clear density for a 12-residue β-strand peptide that originates from the degraded N-terminal jellyroll domain of LptD, suggesting a potential for LptDE inhibition via peptidomimetics. The structure suggests that Oekolampad and similar small siphophages bind to an open state of the LptDE complex. To investigate the SE mechanism, we also determined the cryo-EM structure of the complex of SfLptDE with the SE lipoprotein Rtp45 of phage Rtp (29), which is related to Oekolampad. This structure shows that Rtp45 binds to closed LptDE at the periplasmic OM interface and causes conformational changes in LptD extracellular loops (EL) 4 and 5 that would abolish RBP binding. RBP_Oeko_ binding to SfLptDE and subsequent infection is abolished by several point mutants of LptD. Interestingly, several of these mutants do not appear to increase the sensitivity of *E. coli* for OM stress, suggesting that LptDE functionality is largely preserved and that phage steering via targeting LptDE might be challenging.

## Results

### The Cryo-EM Structure of SfLptDE Contains Bound LptM.

**Fig. 1.**
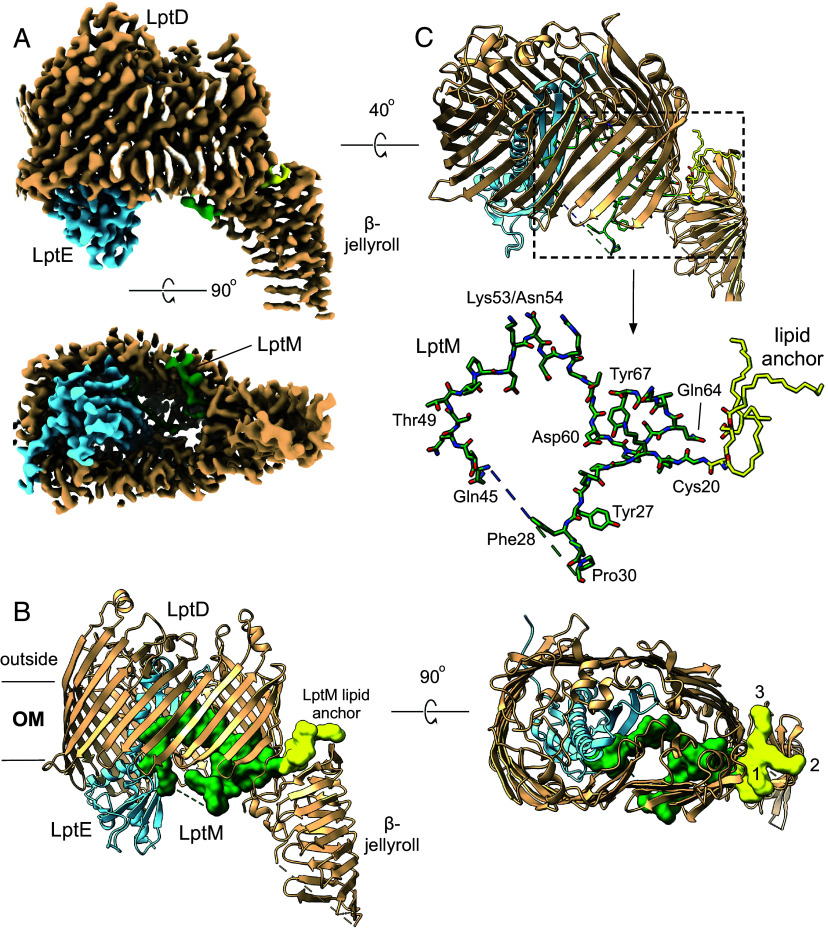
Cryo-EM structure of the LptDE–LptM complex. (*A*) Electron density maps shown at relatively high contour levels, with LptD colored tan, LptE light blue, and LptM dark green. The N-terminal lipid anchor and Cys20 of LptM are yellow. (*B*) Corresponding cartoon models colored as in (*A*) and with LptM in surface representation. In the *Bottom* panel, the acyl chains of the lipid anchor are numbered. (*C*) Stick model of LptM shown within the LptDE complex and without (*Bottom*). The dashed line represents the Ala31-Thr44 segment, which lacks clear density.

We obtained a complex of *Sf*LptE with wild type *E. coli* LptD following expression of the Sf*imp4213* variant (30) of *Sf*LptDE (SfLptD lacking residues 330 to 352 in extracellular loop 4; see *Materials and Methods*, *SI Appendix*, Figs. S1 and S2 and Table S1). The LptDE complexes from *Shigella* and *E. coli* are virtually identical in sequence (three different residues in LptD and 1 different residue in LptE; *SI Appendix*, Fig. S2), and we consider this hybrid structure the same as *Sf*LptDE. We were able to obtain good-quality cryoEM maps around 3 Å resolution (Fig. 1*A* and *SI Appendix*, Fig. S1) without having to resort to fiducial markers such as the Pro-macrobodies used for the recent determination of the cryo-EM structure of LptDE from **Neisseria* gonorrhoeae* (NgLptDE; PDB ID 7OMM) (31). Buildable density is visible for the entire LptDE complex, except for residues Tyr38-Ala67 of LptD and the periplasmic C-terminus of LptE (Ser169-Asn193) (Fig. 1 *A* and *B*). Strikingly, additional density is visible close to periplasmic LptD loops and within the lumen of the complex (Movie S1). Based on the literature as well as on proteomic analysis of the sample, we assigned this density to the small lipoprotein LptM (formerly YifL; Uniprot P0ADN6). This protein was recently identified as a disulfide bond maturation factor of LptD and proposed to mimic bound LPS substrate (32, 33). Density is lacking for the Ala31-Thr44 segment of LptM, likely due to it being in the periplasmic space (Fig. 1*C*), but the assignment of the rest of the protein is unambiguous. Parts of all three acyl chains of the N-terminal lipid anchor are visible, protruding into the DDM micelle via the LptD intramembrane hole previously identified (34), supporting the notion that LptM mimics LPS. The LptM region between Gln45 and Asn54 interacts extensively with LptE, analogous to the proposed interaction of LptE with translocating LPS (*SI Appendix*, Figs. S3 and S4 and Movie S1) (35). Compared to the prediction of bound LptM by Yang et al. (32), the positions of Cys20-Pro30 are very similar to the experimental structure, but beyond Gln45 both structures differ substantially, as evidenced by a difference of ~20 Å between the C-terminal residue Tyr67 (*SI Appendix*, Fig. S3). This is consistent with lower confidence values of the prediction for the C-terminal part of LptM (32). No populations of LptDE complexes lacking LptM were observed.

### Oekolampad RBP Binds to an Open State of the LptDE Complex.

**Fig. 2.**
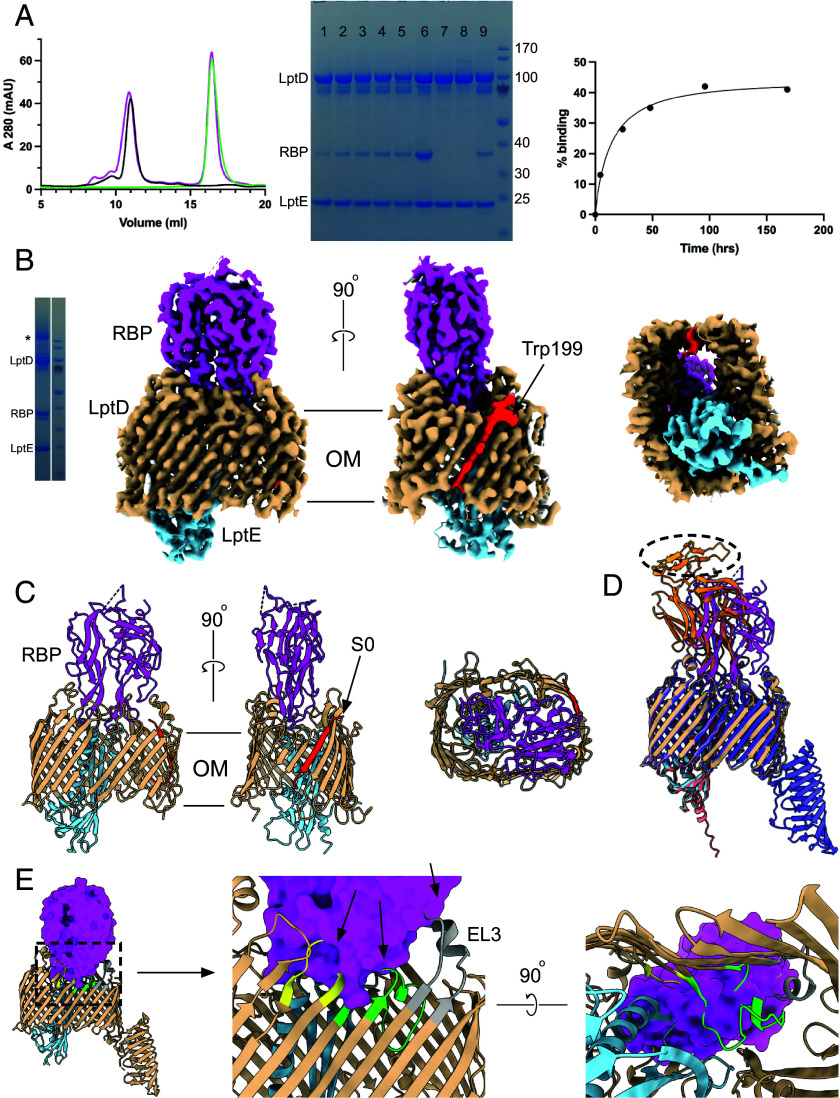
Generation and structure determination of the SfLptDE–RBP_Oeko_ complex. (*A*, *Left*) Representative SEC profiles in DDM for SfLptDE (black), RBP_Oeko_ (green), and the SfLptDE–RBP_Oeko_ complex (magenta; threefold molar excess of RBP). Samples were analyzed after 48 h incubation at 4 °C. (*Middle*) SDS-PAGE (Sodium Dodecyl Sulphate-Polyacrylamide Gel Electrophoresis) samples of a time course of SfLptDE- RBP_Oeko_ complex formation. Lanes 1-5, incubations for 4, 24, 48, 96, and 168 h; lane 6, 1:1 molar ratio of SfLptDE:RBP_Oeko_; lane 7/8, SfLptDE Y671N (R2; 7) and D352Y mutants (R3; 8) incubated with RBP_Oeko_ for 48 h; 9, SfLptDE wild type incubated with RBP_Oeko_ for 48 h (large-scale preparation). To enrich for the complex, the first half of the SfLptDE peak was collected and analyzed (note the slight peak shift in Fig. 2*A*). (*Right*) Percentage RBP_Oeko_ binding generated from the SDS-PAGE samples via band densitometry. LptE was used as the reference band. The amount of complex formation after 48 h varied between 15 to 35%, depending on the LptDE preparation. (*B*) SDS-PAGE and cryo-EM density maps of the SfLptDE–RBP_Oeko_ complex at high contour. The * in the gel indicates disulfide-bonded, dimeric LptD. The rightmost panel shows a view from the periplasmic side. RBP is colored magenta. The bound lateral gate peptide at the front of the complex is red, with the arrow indicating the putative Trp residue. (*C*) Cartoon models of SfLptDE–RBP_Oeko_, with the rightmost panel an extracellular view. (*D*) Comparison between the experimental and AF3-predicted SfLptDE–RBP_Oeko_ structures. RBP_Oeko_ of the AF3 model is colored orange. The hatched oval represents the part of RBP_Oeko_ that is absent or very weak in the cryo-EM density. (*E*) Superposition (on LptD) of the RBP_Oeko_–LptDE complex (LptDE not shown for clarity) with the published LptDE crystal structure (PDB ID 4Q35), showing extensive clashes of RBP with LptDE. The arrows point to parts of LptD EL3-5 that would overlap with bound RBP. EL3 is gray, EL4 lime green and EL5 yellow.

Based on Maffei et al., we initially focused on the RBP of the coliphage Rtp, named Rtp44, which was proposed to bind LptD as its terminal receptor based on its genomic location and homology (96% sequence ID) to the LptD-targeting AugustePiccard (bas01) RBP (28). However, *E. coli* expression of this protein failed. Switching targets, we obtained high expression levels for C-terminally His6-tagged RBP from Oekolampad (bas018; genus *Dhillonvirus*), whose receptor was also identified as LptD by Maffei et al. RBP_Oeko_ has 33% sequence identity to Rtp44 and a very similar predicted fold (rmsd 1.1 Å for 244 Cα pairs out of 309 total; *SI Appendix*, Fig. S5*A*). Moreover, Black et al. predicted models of RBPs from diverse LptD-dependent phages, including the putative RBP from OzMSK, revealing significant structural homology despite low sequence identity (36), suggesting a similar binding mechanism among LptD-targeting RBPs. Incubation of dodecyl-maltoside (DDM)-purified SfLptDE with ~threefold molar excess of RBP_Oeko_ resulted in the slow formation of a complex as judged by size-exclusion chromatography (SEC) in DDM (Fig. 2*A*). Given that complex formation is not quantitative, a larger-scale 48-h co-incubation was set up using tag-less SfLptDE (*Materials and Methods*) and His-tagged RBP_Oeko_. The complex was purified via IMAC and SEC (Fig. 2*B*), and cryo-EM data were collected. Data processing gave two major particle classes (*SI Appendix*, Table S1), the largest of which corresponded to a monomer and the other to a side-by-side dimer (*SI Appendix*, Fig. S6). Contrasting with the native LptDE(M) structure described above, no density was observed for the LptD jellyroll domain in both classes, despite most of LptD being intact (Fig. 2 *A* and *B*).

The monomeric particle class gave maps to ~2.8 Å resolution and showed clear density for RBP_Oeko_ bound to the extracellular face of LptDE (Fig. 2*B*). RBP_Oeko_ inserts deeply into the LptDE lumen with a total interface area of almost 1,700 Å^2^ as analyzed via PISA (37), with a complex formation significance score (CSS) of 1.00. RBP_Oeko_ forms 20 hydrogen bonds and two salt bridges with LptD and two hydrogen bonds with LptE, one of which is between main-chain atoms (*SI Appendix*, Fig. S7*A*). Thus, despite the very slow rate of complex formation, once established the interaction between LptDE and RBP_Oeko_ is likely to be tight. Most polar interactions of RBP_Oeko_ are with LptD EL3-5 and EL11 (*SI Appendix*, Fig. S7*A*). The AlphaFold3 (AF3) (38) prediction of RBP_Oeko_ in isolation is virtually identical to that in the complex structure (Cα rmsd 0.75 Å), suggesting that binding to LptDE causes very limited structural changes in RBP_Oeko_. Despite this, the prediction of the SfLptDE–RBP_Oeko_ complex shows a substantially different interaction, with RBP_Oeko_ shifted and inserted less deeply (Fig. 2*D*). The part of RBP_Oeko_ that would be proximal to the rest of the phage tail is very poorly ordered, an intriguing similarity to the phage T5 RBP (pb5) bound to the FhuA receptor (PDB ID 8A8C) (3). A superposition of LptDE structures shows that RBP_Oeko_ could not bind to the crystallized, “resting state” LptDE complex due extensive overlaps (Fig. 2*E*). Therefore, RBP_Oeko_ binds to a SfLptDE state with a substantially different conformation compared to hitherto determined experimental structures, which are very similar to AlphaFold predictions.

Strikingly, strong density for an extra β-strand peptide is visible between LptD strands S1 and S26, i.e., it is bound in the lateral gate (Fig. 2 *B* and *C*). Based on distinctive density indicating a Trp at the extracellular OM interface (Fig. 2*B*), we assigned the peptide as LptD residues Glu197-Pro209, corresponding to βN10 of the jellyroll domain (*SI Appendix*, Fig. S8), and named it S0. The peptide is oriented parallel to S26 and forms a total of 14 hydrogen bonds with strands S1 and S26, with all but one of them between backbone peptide bonds (*SI Appendix*, Fig. S7*B*). The presence of the peptide means that RBP-bound LptD has a 27-stranded barrel. The distance between the backbone atoms of strands S1 and S26 is ~10 Å, which would likely be large enough to allow lateral passage of an LPS molecule into the OM. Molecular dynamics (MD) simulations in a model *E. coli* OM showed that all four components of the complex are stable (*SI Appendix*, Fig. S9). Interestingly, removal of the bound S0 strand (while keeping RBP_Oeko_ bound) resulted in a very rapid closing of the lateral gate and the formation of several stable interstrand hydrogen bonds (*SI Appendix*, Fig. S10), suggesting that the barrel has a high plasticity.

To exclude the possibility that the formation of the LptDE–RBP_Oeko_ complex was a one-off adventitious event, we generated another cryo-EM sample using different LptDE and RBP_Oeko_ preparations. Data collection and processing (dataset 2; *SI Appendix*, Fig. S11 and Table S1) resulted in an identical structure of the LptDE–RBP_Oeko_ complex (Cα rmsd 0.4 Å), including the bound S0 strand. Interestingly however, when contoured at low levels, this map shows continuous density connecting the S0 lateral gate peptide to LptD strand S1 (*SI Appendix*, Fig. S12 and Movies S2 and S3), confirming the assignment of strand S0 as residues 197 to 209 of the N-terminal LptD jellyroll domain.

**Fig. 3.**
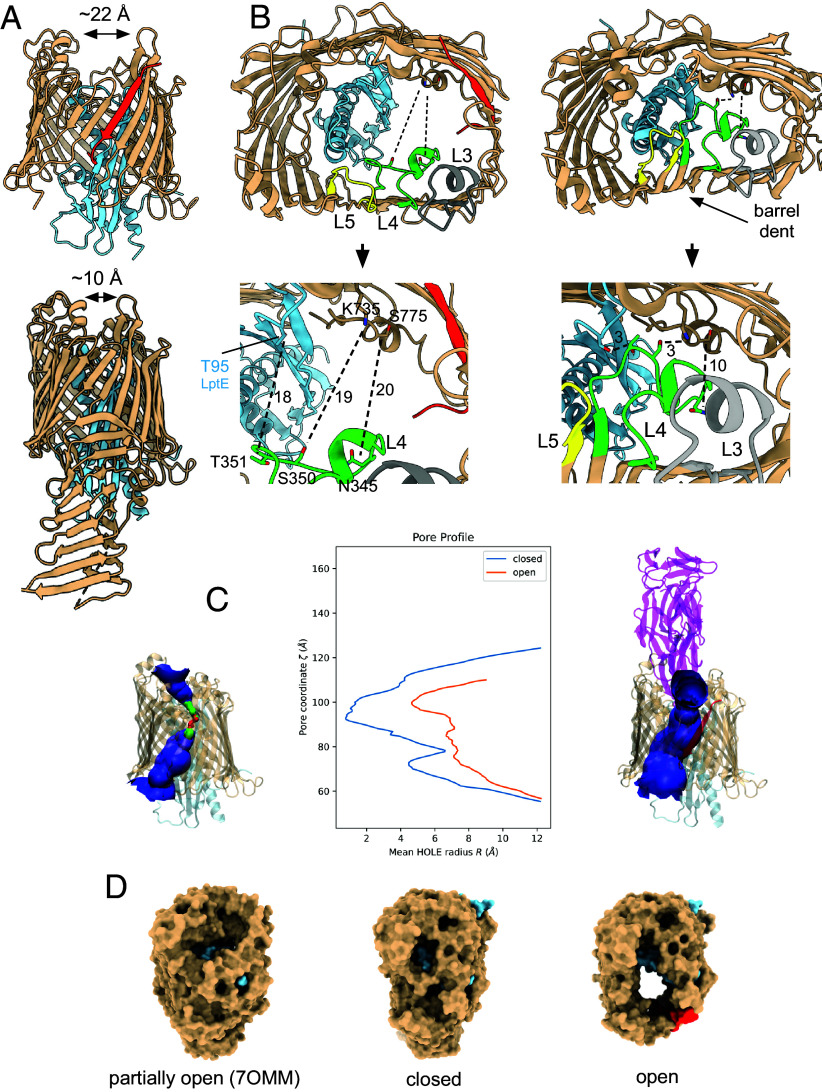
An open state of the LptDE translocon. (*A*) Front views of open (*Left*) and closed LptDE. The lateral gate peptide is in red. RBP_Oeko_ is not shown in the *Left* panel for clarity. The arrows indicate the distance between loops on both sides of the barrel. (*B*) Extracellular views of open (*Left*) and closed SfLptDE. EL3 (Tyr291-Trp311), EL4 (Lys333-Thr356), and EL5 (Phe377-Ser389) are colored gray, lime green, and yellow, respectively. Dashed lines indicate distances discussed in the text. The *Bottom* panels show zoomed-in views of the same area, with distances given in Å. Views were generated from LptD superpositions. (*C*) HOLE (39) surface representations of resting state LptDE (PDB 4Q35; jellyroll domain not shown) on the left and SfLptDE-Oeko_RBP_ on the right. Channel surfaces connecting the periplasmic and extracellular space are shown in blue (green, diameter < 3 Å; red, diameter < 1.5 Å). The structural models are approximately aligned to the pore profiles. (*D*) Extracellular surfaces for (*Left*) partially open NgLptDE [PDB ID 7OMM; (31)] and the closed and open states of SfLptDE. Views generated from LptD superpositions.

According to the consensus model, LPS insertion into the OM requires not only opening of the lateral gate for diffusion of the lipid A acyl chains but also a channel to the extracellular side to allow passage of LPS polar groups such as core oligosaccharides and the O-antigen. As may be expected from the insertion of an additional β-strand, the “laterally open” LptD barrel is much wider at the front of the complex (Fig. 3*A*), with strands β1-β12 and EL1-EL5 displaced outward by up to 7 Å. As a result, the pronounced dent in the closed barrel between strands 5-10 has disappeared in the open state, and the barrel is almost rectangular in shape (Fig. 3*B* and Movie S3). The strands and EL6-EL9 at the back of the complex are virtually identical, but, moving toward the front, the (outward) backbone shifts become again pronounced and reach 6 Å for EL11-13 and the strands connecting them. Overall, the Cα RMS values between the open and closed state are 2.7 Å for LptD and 1.2 Å for LptE. The dramatic structural changes between both states are illustrated by the backbone hydrogen bond between Thr351 in LptD EL4 and Thr95 of LptE in the closed state. In the open state, the Cα atoms of these residues are ~18 Å apart. As another example, the hydroxyl of Ser350 in EL4 makes a hydrogen bond with the side chain of Lys735 in β25/EL13 in closed LptD but is separated by ~19 Å in the open state, with Ser350 now hydrogen bonding to Gln299 of RBP_Oeko_. The Cα atoms of Asn345 in EL4 and Ser775 close to the LptD C-terminus move from ~10 Å in the closed state to ~20 Å in the open state (Fig. 3*B*). As a result of the conformational changes, the open state has a wide channel connecting the periplasmic space to the extracellular environment with a minimum diameter of ~10 Å (Fig. 3*C*), wide enough to allow passage of hydrophilic LPS moieties. No such channel is present in the partially open structure of **N.* gonorrhoeae* LptDE and in closed LptDE complexes (Fig. 3*D*) (20, 22, 23, 31, 33) and the minimum pore diameters of the latter are likely too small to allow significant water passage.

Besides the main particle class for the LptDE–RBP_Oeko_ complex, dataset 1 contains a smaller class of LptDE dimers for which we could generate maps to ~3.4 Å (*SI Appendix*, Fig. S6 and Table S1). Interestingly, only one of the LptDE complexes has bound RBP_Oeko_ and represents the open state, whereas the other lacks the RBP and is closed. No particle classes were observed for dimeric open or closed complexes. The LptD barrels are stacked front-to-front, with complex formation likely driven by an intermolecular disulfide bond between the Cys725 residues of both LptD protomers (Fig. 2*B* and *SI Appendix*, Fig. S13). Given that the N-terminal jellyroll domains would clash with each other this dimeric complex is clearly nonphysiological, but it allows a good appreciation of the differences between the open and closed states of the LptDE complex. Neither the open nor the closed state shows any density for LptM, presumably due to the lack of the jellyroll domain.

### Spontaneous LptD Mutants Abolish *E. coli* Infection by Oekolampad.

**Fig. 4.**
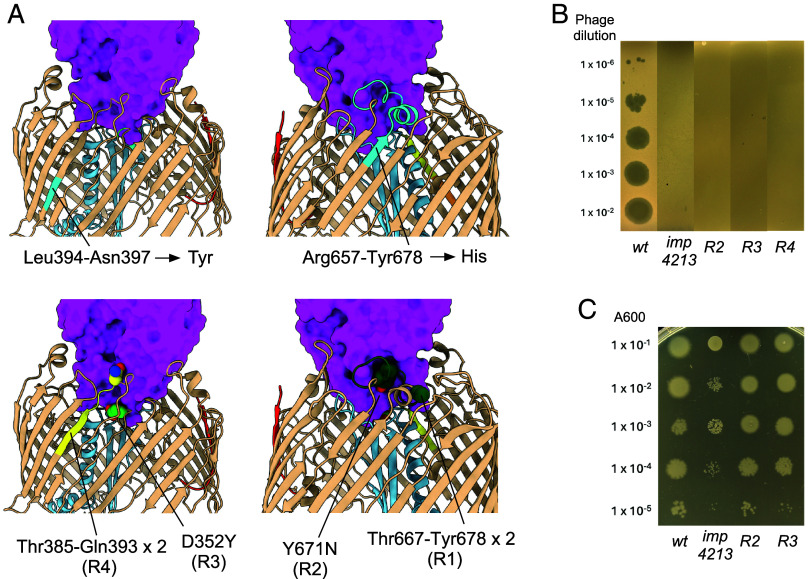
Characterization of Oekolampad-resistant *E. coli* mutants. (*A*) Locations of the resistant mutants generated by Maffei et al. (*Top* panels; mutated regions shown in cyan) and in the current study. Point mutants and duplication insertion sites are shown in space filling models, and the RBP surface is colored magenta. Mutated EL4 residues are colored lime green, EL5 residues yellow, and EL11 residues dark green. (*B*) Oekolampad phage dilutions spotted on soft agar LB (Luria-Bertani) plates with *imp4213* and Bl21(DE3) strains. (*C*) Strain dilutions spotted on LB plates with 0.5% SDS and 0.5 mM EDTA (ethylenediaminetetraacetic acid).

Maffei et al. identified two mutations in LptD that conferred resistance to a panel of LptD-targeting siphophages, including Oekolampad (Bas18). The first of these was a 4-residue deletion (Leu394-Asn397) in strand β10 that was replaced by a tyrosine residue, and the second was a 22-residue deletion (Arg657-Tyr678) in EL11 replaced by a histidine. The effect of the first deletion is hard to predict given its location within the OM-embedded part of the barrel, but it most likely affects EL5 which interacts with RBP (Fig. 4). The effect of the second mutation is easier to rationalize, given the removal of virtually the entire RBP-interacting EL11. Thus, for both Maffei mutants the generation of phage resistance is rationalized by the LptDE–RBP_Oeko_ structure. Notably, this EL11 deletion mutant was also recently obtained in a separate study of LptD-targeting phages, confirming its ability to block infection (36).

We were interested to further define the “resistance-space” of LptD and to this end characterized four additional resistant mutant *E. coli* Bl21(DE3) strains that spontaneously grew on Oekolampad-infected soft agar plates (*Materials and Methods*). Gratifyingly, all four strains had changes in their LptD as determined via whole-genome sequencing. Two mutants (R1 and R4) had insertions that both consisted of duplications. In R1, the duplicated sequence Thr667-Tyr678 in EL11 (12 residues) has inserted at Ala666, and in R4 the 9-residue sequence Thr385-Gln393 in EL5 has inserted at Asn384 (Fig. 4). Strikingly, the other two mutants (R2 and R3) are LptD point mutations: Y671N in EL11 (R2) and D352Y in EL4 (R3). All four mutants are clearly resistant to Oekolampad infection on agar plates (Fig. 4*B*). Intriguingly, similar mutations have recently been identified in unrelated LptD-targeting phage systems. A study of resistance evolution in *E. coli* exposed to the JNUWD phage revealed three LptD point mutations, Y671D, D226Y, and D352Y, that conferred phage resistance (40). Notably, two of these mutants (Y671D and D352Y) concern the same residue as our R2 and R3 mutants (and R3 is identical). These independent findings emphasize the key role of EL11 and EL4 in phage binding and suggest that certain sites within LptD act as evolutionary “hotspots” for resistance.

Together with the Maffei variants, these data show that resistance is readily generated via mutation of the phage receptor, even for an essential OMP complex. Given the large LptDE–RBP_Oeko_ interface and the high number of intermolecular hydrogen bonds, the generation of resistance by just a single point mutation is remarkable. To confirm this result in vitro and to ascertain whether the resistance is due to a defect in RBP_Oeko_ binding to LptDE, we generated the R2 and R3 point mutants in the pBAD22 expression vector and purified the LptDE complexes. Following incubation with RBP_Oeko_ as done for wild type LptDE, no copurifying RBP_Oeko_ was observed for both mutants (Fig. 2*A* and *SI Appendix*, Fig. S14). These results show that both point mutants abolish or weaken the interaction of RBP_Oeko_ with LptDE and suggest this causes the observed resistance against infection. Interestingly, the *imp4213* strain [lacking the entire EL4; residues 330 to 352; (30)] is also resistant to infection, demonstrating that specific interactions of RBP_Oeko_ with EL4 are required for complex formation. To determine whether the resistance mutations affected OM permeability (potentially resulting from LptDE malfunction), we determined the sensitivity of the point mutant strains R2 and R3 toward SDS/EDTA on solid media. Both mutants behaved like the wild type whereas the *imp4213* control strain was, as expected, sensitive to OM stress (Fig. 4*C*). These findings are consistent with a recently reported EL11 deletion in EcLptDE, which conferred phage resistance without detectable effects on bacterial growth or recombinant protein expression (36). We conclude that LptDE can readily mediate resistance toward binding by small siphophages without a major loss of fitness in rich medium, indicating that phage steering via LptDE may be challenging, at least for these phages.

### Mechanism of SE of LptDE-Targeting Small Siphophages.

**Fig. 5.**
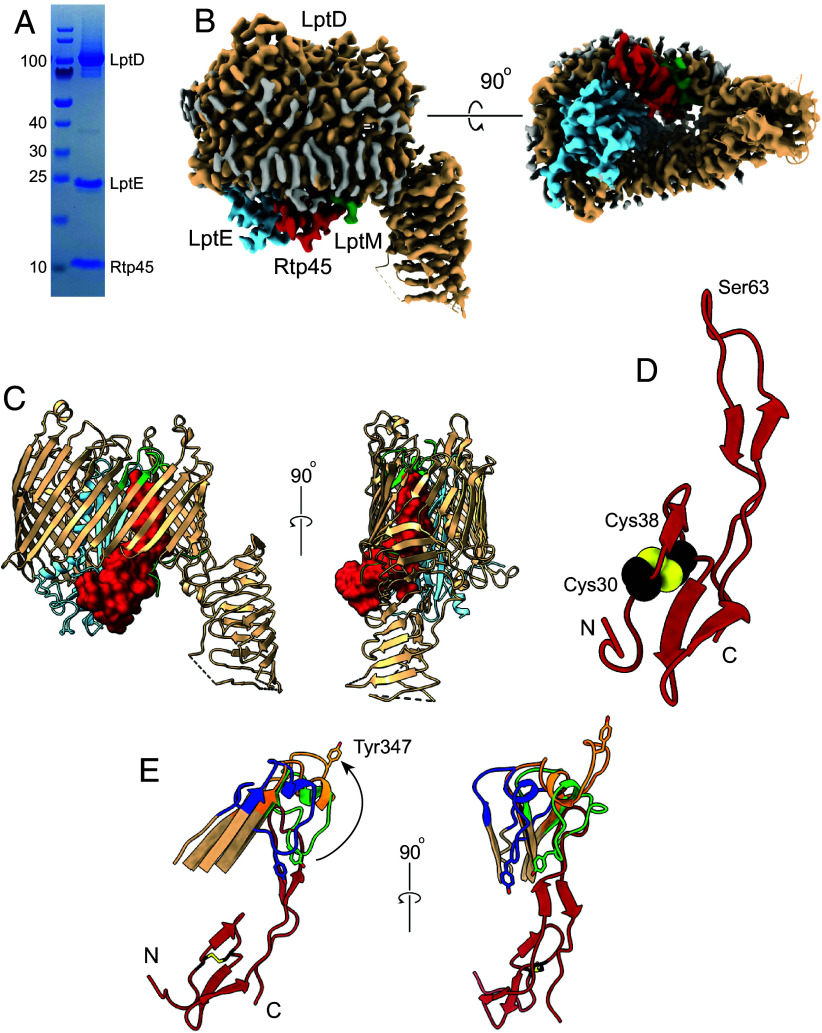
SE mechanism of phages Oekolampad and Rtp. (*A*) SDS-PAGE gel of the SfLptDE–Rtp45 complex following SEC. (*B*) Cryo-EM density maps of the SfLptDE–Rtp45 complex viewed from the left-hand side and from the periplasmic space. Rtp45 is colored maroon and is sandwiched between LptE and LptM. (*C*) Cartoon viewed from the left-hand side and from the front, with Rtp45 shown in surface representation. LptD EL4 is lime green. (*D*) Cartoon model of Rtp45 viewed in the same orientation as in C (*Left*-hand panel), with Cys30 and Cys38 shown as space-filling models. (*E*) Cartoon models viewed from the left-hand side and from the front (*Right* panel) of EL4 in LptDEM (lime green), LptDE–RBP (open state, blue), and LptDEM-Rtp45 (orange, with Rtp45 in maroon). The movement of Tyr347 in EL4 from the closed LptDEM state to the Rtp45-bound state is indicated with the arrow. The reverse movement would be blocked by Rtp45, providing a rationale for abolishing RBP binding. Views generated from LptD superpositions.

Bacteriophages produce SE factors that prevent nonproductive phage absorption to already infected cells and to cell fragments produced post lysis. With phage adsorption to (outer) membrane receptors, this amounts to an effect by the SE factor that prevents RBP binding to the receptor. For the TonB-dependent transporter (TBDT) FhuA, the OM receptor for coliphage T5, the SE lipoprotein Llp binds to the periplasmic face of FhuA and induces “reverse” allosteric conformational changes in extracellular loops that abolish binding of the T5 RBP (3). However, since LptDE is not a TBDT it is not clear how periplasmic SE lipoproteins could abolish RBP binding to LptD. To answer this question, we initially tried to express the Oekolampad SE lipoprotein (bas18_0026; SE_Oeko_) in *E. coli* but failed. Good expression was obtained for the SE lipoprotein Rtp45 from phage Rtp (UniProt ID: Q333D9), which has 35% sequence identity to SE_Oeko_ (*SI Appendix*, Fig. S5). We generated the SfLptDE–Rtp45 complex via co-overexpression in *E. coli* (Fig. 5*A*). The cryo-EM data contain only one major particle class, with bound Rtp45 (*SI Appendix*, Fig. S15 and Table S1). The small lipoprotein (~6 kDa) is bound to the periplasmic, left-hand side of LptDE close to β-strands 5-8 (Fig. 5 *B* and *C*). Except for the N-terminal three residues including the lipid anchor, the entire protein is visible in the map. PISA analysis shows that the LptD–Rtp45 interface has an area of almost 1,000 Å^2^ (CSS = 1.00). There are 17 polar interactions between Rtp45 and LptD (*SI Appendix*, Fig. S16). A β-sheet is present between Rtp45 residues 30 to 35 and LptD residues 318 to 323 at the base of strand β6. In addition, Rtp45 forms two hydrogen bonds with LptE on one side and two hydrogen bonds with LptM on the other (*SI Appendix*, Fig. S16), meaning it is sandwiched between both lipoproteins (Fig. 5*B*). Only the first ~10 residues of LptM are visible in this map, similar to other LptDEM structures (Fig. 5 *B* and *C*). The fact that LptM is bound in the presence of Rtp45 must mean that the remainder of LptM (i.e., the part not visible in the map) is in the periplasmic space and supports the notion that LptM might be permanently bound to the LptDE translocon via its N-terminal ~10 residues. Besides a disulfide bond between Cys30 and Cys38, Rtp45 has a long, partially β-stranded hairpin that protrudes upward through the LptD lumen (Fig. 5*D*). As is common for phage proteins, being underrepresented in structural databases, the AF3 prediction of Rtp45 is very different from the experimental structure, although the Cys30-Cys38 disulfide is correctly predicted (*SI Appendix*, Fig. S5*B*). Interestingly, the model of SE_Oeko_ is more similar to the Rtp45 experimental structure, even though the predicted disulfide is incorrect as it involves the N-terminal cysteine. Despite this, it seems a reasonable assumption that the interaction of SE_Oeko_ with LptD is similar to that of Rtp45. The AF3 predictions for the LptDE–SE_Oeko_ and LptDE–Rtp45 complexes all show the SE proteins interacting with the LptD jellyroll domain (*SI Appendix*, Fig. S17) and are clearly incorrect.

The main consequence of Rtp45 binding is that EL4 of LptD is displaced toward the extracellular side by up to 20 Å (for the Tyr347 Cα atom; Fig. 5*D*). This conformation of EL4, together with the presence of bound Rtp45 will cause SE due to i) induction of a LptD conformation that is incompatible with RBP binding and ii) blocking the movement of EL4 in the open state (Fig. 5*E* and Movie S4). Thus, the conformational changes in LptD that block binding of the phage RBP result from physical displacement of extracellular loops by the SE protein, and not from long-range allostery as in FhuA.

## Discussion

The most important and surprising finding of our work is that the RBP of the small siphophage Oekolampad, and most likely related phages, binds to an open state of the LptDE translocon, enabling experimental visualization. The formation of the complex is extremely slow, suggesting that open LptDE states enabling RBP_Oeko_ binding are very rare and/or short-lived in vitro, which is consistent with existing structural data for LptDE. The fact that MD simulations show that the lateral gate in LptDE–RBP_Oeko_ rapidly closes upon removal of the bound jellyroll-derived S0 segment in silico suggests the barrel is flexible and raises the possibility that bound RBP would not interfere with LPS translocation. Interestingly, very low levels of complex appear to be formed between LptDE lacking the jellyroll domain (LptD_Tr_E) and RBP_Oeko_ (*SI Appendix*, Fig. S14), suggesting that S0 binding favors complex formation, presumably by stabilizing the open state, but is not absolutely required.

We analyzed the purified LptDE–RBP sample and identified hundreds of peptides (*Materials and Methods*), with those from LptD originating exclusively from the N- and C-terminus (Dataset S1). Peptides with part of the S0 sequence are present but occur with low frequency (*SI Appendix*, Fig. S18), supporting our notion that it is still attached to the majority of degraded LptD molecules. The degradation of the LptD jellyroll domain required for formation of the open, RBP binding-competent LptDE state in vitro are likely consequences of the detergent extraction and purification procedures. However, the high specificities of receptor–RBP interactions suggest that the visualized open state is relevant in vivo. Indeed, translocating LPS molecules in vivo should induce a state of LptDE with similar properties as the one in our structures, i.e., with an open lateral gate and a large channel connecting the periplasmic space to the extracellular side. We therefore propose that Oekolampad and similar phages target an open conformation of the LptDE translocon that might correspond to an active state, perhaps to ensure efficient phage replication via selecting growing cells. An interesting implication of the bound lateral gate peptide is that it suggests that LptDE function might be inhibited by peptidomimetics that bind stably to the open lateral gate, such as those discovered for the BamA component of the BAM complex (41, 42). To begin to test this hypothesis we synthesized the S0 peptide (LptD residues 196 to 207) and determined MIC values against a panel of *E. coli* strains, most of which have compromised OM barriers. Intriguingly, while no MICs were observed for most of the strains, both the L- and D-isomer of the S0 peptide generated weak MICs against the *imp4213* strain (*SI Appendix*, Table S2), supporting the structural data that the S0 interaction mainly involves backbone interactions (*SI Appendix*, Fig. S7*B*). However, it is worth noting that this mutant is generally more susceptible to nonspecific, membrane-disrupting antimicrobial peptides due to its stalling on the BAM complex (43), which may contribute to the observed activity. Whether or not effective antibiotics targeting the LptD lateral gate can be designed remains an open question; thus far, only peptidomimetics that target periplasmic parts of *P. aeruginosa* LptDE have been obtained (44, 45). However, the availability of an open state that is likely to be populated in vivo may open up opportunities for in silico drug discovery of LptD inhibitors.

The presence of LPS-mimicking LptM within the purified LptDE complex is very intriguing. The density for the hitherto unobserved C-terminal half of LptM is weaker than that for LptDE, implying mobility or unresolved alternate conformations. However, the density for the first N-terminal LptM residues is comparable to that of the neighboring LptD barrel, suggesting LptM is bound to at least the majority of LptDE complexes within the sample. Given that LptDE does not copurify with LPS, it is unlikely that LptM binds to LptDE during detergent extraction. LptM must therefore be present within fully matured, functional LptDE complexes in the OM. Based on our structure, LptDE-bound LptM might block LPS transport, raising the question of how the presence of bound LptM is compatible with normal cell growth. This question is very relevant given that LptM is present in a serendipitously isolated hybrid complex between overexpressed, His-tagged SfLptE, and endogenous EcLptD (*Materials and Methods*). Thus, the observed LptD–LptM interaction is not an artifact of LptD overexpression. Some insights into this conundrum may be gained from a quantitation of the *E. coli* proteome (46), which showed that LptD and LptE are present at ~400 to 600 copies per cell depending on the strain and growth media. By contrast, the ABC transporter components LptC, LptF, and LptG are much less abundant with ~60 to 120 copies per cell, whereas LptM is very abundant (~2,000 to 2,500 copies/cell). Thus, based on these numbers, only a minor fraction of LptDE molecules can be active at any time, i.e., physically engaged within a periplasmic protein bridge. If, following biogenesis, all LptDE complexes contain LptM and the intermembrane Lpt protein bridges are very stable, it is possible that LptM is cleared from its binding site by translocating LPS molecules in only a small fraction of the LptDE pool, which might be hard to detect in a cryo-EM dataset. An interesting implication of this hypothesis, supported by a recent study (33), is that LptDE may mediate surface exposure not only of LPS but also of LptM and perhaps other small lipoproteins like its homologue in *Borrelia burgdorferi* (47). Whether this has functional significance is not clear, but it would explain, for example, why part of the Braun’s lipoprotein (Lpp) pool is surface exposed in *E. coli* (33, 48). Alternatively, our data might indicate that LptM is an integral component of the LptDE translocon, perhaps to assist LPS translocation, a notion supported by recent data that suggest LptM binding weakens interactions between S1 and S26 of the lateral gate (32). Although our LptDEM structure suggests that the part of LptM that is present in the LptDE lumen would block the LPS translocation pathway, this blockage could be relieved by movement of this part of LptM into the periplasmic space while remaining bound to the translocon via its N terminus, as observed in the LptDE–Rtp45 complex (Fig. 5) and other recent structures (32).

## Materials and Methods

### Cloning and Mutagenesis.

The pBAD22 plasmid containing **S.* flexneri* lptD and His6-tagged lptE (20) was generously provided by the Huang lab (Institute of Biophysics, Chinese Academy of Sciences). Truncation of lptD (Δ26-201) and the insertion of a TEV protease recognition site immediately upstream of the C-terminal His-tag on lptE were performed using site-directed mutagenesis with the Q5 High-Fidelity DNA polymerase kit (New England Biolabs), following the manufacturer’s protocol. Mutagenic primers were designed using the NEBaseChanger tool. The full-length genes encoding for Rtp44, RBP_Oeko_, RTP45, and SE_Oeko_ were synthesized by Eurofins Genomics, and cloned into the pET28b expression vector (Novagen) via *Nco*I and *Xho*I sites, appending the sequence LEHHHHHH to the proteins. Genomic DNA was isolated from spontaneous phage resistant **E. coli* lptD* mutants R1-R4 via the Sigma-Aldrich GenElute bacterial gDNA kit and sent for whole genome sequencing (Eurofins genomics). The *lptD* genes from the resistant mutants were amplified via PCR using primers that incorporated *Nco*I and *Hind*III restriction sites at the 5′ and 3′ ends, respectively. Both amplified insert and SfLptDE-pBAD22 vector were digested with *Nco*I and *Hind*III (New England Biolabs), PCR purified, and ligated using T4 DNA ligase (New England Biolabs). The mutants are the following: R1, Thr667-Tyr678 duplication at Ala666; R2, Y671N; R3, D352Y; R4, Thr385-Gln393 duplication at Asn384.

### Protein Expression and Purification.

#### LptDE(M) and mutants.

Full-length (LptDE), truncated (LptD_Tr_E), and mutant variants (*imp4213*, *R2,* and *R3*) of LptDE were moved into electrocompetent *E. coli* Bl21(DE3)Δcyo cells (ΔcyoB, truncated *cyoA* and *cyoC*) via electroporation. Overnight starter cultures were grown in LB medium supplemented with ampicillin (100 μg/mL) and used to inoculate 1 L flasks of LB (100 μg/mL ampicillin) to a starting OD_600_ of ~0.05. Cultures were grown at 37 °C, 180 rpm until OD_600_ reached 0.8 to 1.0, at which point expression was induced with 0.1% (w/v) arabinose. Full-length LptDE and its mutants were expressed at 37 °C, 180 rpm for 2.5 h postinduction, whereas LptD_Tr_E was expressed at 18 °C and 150 rpm for 20 h postinduction. For coexpression of LptDE and Rtp45, cells were sequentially transformed with SfLptDE-pBAD22 and Rtp45-pET28. Coexpression was performed under the same conditions as full-length LptDE; no IPTG was added to express Rtp45, as leaky expression from the T7 promoter was sufficient to produce excess Rtp45 relative to LptDE.

Cells were harvested by centrifugation at 4 °C for 20 min at 4,200 rpm (Beckman J6-HC, JS-4.2 rotor), and resuspended in TBS (20 mM Tris-HCl, pH 8.0, 300 mM NaCl) supplemented with DNase I. Cells were resuspended with a Dounce homogenizer, followed by two passes through a Constant Systems cell disruptor (0.75 kW model) at 20,000 psi. Lysates were clarified by ultracentrifugation (42,000 rpm, 50 min, 4 °C; Beckman Optima XE-90, 45Ti rotor), and the membrane pellet was resuspended in TBS containing 2% (w/v) LDAO. After homogenization and stirring for 1 h at 4 °C, insoluble material was removed by ultracentrifugation (30,000 rpm, 30 min, 4 °C; Beckman Optima XE-90, 45Ti rotor).

The supernatant containing solubilized LptDE was loaded onto a gravity-flow IMAC column packed with Ni^2+^-charged Chelating Sepharose (Cytiva), pre-equilibrated in wash buffer (20 mM Tris-HCl, pH 8.0, 300 mM NaCl, 30 mM imidazole) + 0.15% (w/v) DDM. The column was washed with 30 column volumes of wash buffer. Bound protein was eluted with 3 column volumes of elution buffer (20 mM Tris-HCl, pH 8.0, 300 mM NaCl, 200 mM imidazole) + 0.15% (w/v) DDM. The eluate was concentrated via a 100 kDa MWCO Amicon Ultra centrifugal filter (Millipore) and loaded on a Superdex-200 16/600 s column (Cytiva), equilibrated in 10 mM HEPES (4-(2-hydroxyethyl)-1-piperazineethanesulfonic acid), 100 mM NaCl, and 0.03% DDM. When required (e.g., for removal of excess LptE), an analytical SEC column (Superdex 200 Increase 10/300 GL column; Cytiva) was run next, with the same buffer as above. Complexes for cryoEM were concentrated to 5 to 8 mg/mL. Yields of wild type and R2/R3 mutants of SfLptDE were 0.5 to 1 mg/L cell culture.

When we carried out the above expression and purification procedures for a pBAD22-encoded *imp4213* variant of SfLptDE, we obtained low amounts of LptD and, as usual, an excess of LptE after IMAC. Following two rounds of SEC we obtained a reasonably well-defined peak that was concentrated, and data were collected by cryoEM. Upon inspection of the maps, it became evident that, instead of the *imp4213* variant lacking EL4, we had obtained a complex between SfLptE and EcLptD (*SI Appendix*, Fig. S1). Presumably, expression levels of *imp4213* were very low and/or the protein was unstable during purification. Given that EcLptD and SfLptD are virtually identical (differences are L_Ec_453F_Sf_, R_Ec_657H_Sf_, W_Ec_691R_Sf_ in LptD and T_Ec_190 M_Sf_ in LptE) we did not pursue structure determination of a “true” wild type SfLptDE complex.

TEV cleavage of SfLptDE was carried out only when necessary (i.e., for isolation of the LptDE–RBP_Oeko_ complex) since it consistently reduced overall protein yield and the required reducing conditions could disrupt LptD disulfide bonds. Following initial IMAC purification, samples were concentrated and diluted at least 20-fold into cleavage buffer [50 mM Tris-HCl, pH 8.0, 0.5 mM EDTA, 0.2 mM TCEP, 0.1% (w/v) DDM]. Protein concentration was determined by absorbance at 280 nm, and His-tagged TEV protease was added at a ratio of 1 mg per 5 mg of protein. The digestion was carried out overnight at 4 °C. If present, precipitate containing excess LptE was removed by centrifugation, and the supernatant was adjusted to 250 mM NaCl and 10 mM imidazole before being applied to a second IMAC column to remove TEV protease, uncleaved LptDE, and His-tagged fragments. Flowthrough and wash fractions were concentrated and applied to a Superdex 200 Increase 10/300 GL column (Cytiva) equilibrated in 10 mM HEPES, pH 7.5, 100 mM NaCl, 0.05% (w/v) DDM. Peak fractions were pooled, concentrated, and flash-frozen in liquid nitrogen for storage at –80 °C.

#### RBP_Oeko_.

RBP_Oeko_-pET28 plasmid was transformed into electrocompetent *E. coli* Bl21(DE3) cells. Overnight cultures grown in LB with kanamycin (35 μg/mL) were used to inoculate 1 L flasks (35 μg/mL kanamycin, starting OD_600_ ~ 0.05). Cultures were grown at 37 °C, 180 rpm, until OD_600_ reached 0.4 to 0.6, then chilled at 4 °C for 1 h prior to induction with 400 μM IPTG. Expression proceeded for 20 h at 18 °C, 150 rpm. Cells were harvested and lysed as above, and clarified by centrifugation (19,000 rpm, 30 min, 4 °C; Beckman JA-25.50 rotor). The supernatant was applied to a gravity-flow IMAC column packed with Ni^2+^-charged Chelating Sepharose (Cytiva) pre-equilibrated in wash buffer. After washing with 30 column volumes of wash buffer, protein was eluted with 3 column volumes of elution buffer and concentrated using a 30 kDa MWCO Amicon Ultra centrifugal filter (Millipore). The protein was further purified by SEC using a Superdex 200 Increase 10/300 GL column equilibrated in 10 mM HEPES, pH 7.5, 100 mM NaCl, 10% glycerol. Peak fractions were pooled, concentrated, and flash-frozen in liquid nitrogen for storage at –80 °C. Yields were ~10 to 15 mg/L cell culture.

### DE–RBP Complex Formation, Purification, and Time Course Analysis.

To assess the formation kinetics of the DE–RBP complex, purified SfLptDE and RBP_Oeko_ were mixed at a 1:3 molar ratio (DE:RBP) in buffer containing 10 mM HEPES, pH 7.5, 100 mM NaCl, and 0.05% (w/v) DDM. The sample was incubated at 4 °C, and aliquots were withdrawn at 4.5, 24, 48, 96, and 168 h. Each timepoint sample was subjected to size-exclusion chromatography using a Superdex 200 Increase 10/300 GL column (Cytiva) equilibrated in 10 mM HEPES, pH 7.5, 100 mM NaCl, 0.05% (w/v) DDM. The leading half of the major elution peak was collected, concentrated, and analyzed by SDS-PAGE after adding 4× SDS-PAGE sample buffer and 5 mM DTT, using 4 to 12% NuPAGE Bis-Tris gels and MES running buffer (Thermofisher Scientific). Band intensities were quantified using ImageJ software. The ratio of RBP to LptE band intensity was plotted over time to assess complex formation. Based on the time course analyses, large-scale tag-less LptDE and tagged RBP_Oeko_ incubations for cryoEM were typically purified after 48 h of incubation. Following incubation with RBP_Oeko_ at a 3:1 molar ratio (RBP:LptDE), the sample was applied to a gravity-flow IMAC column equilibrated in wash buffer + 0.15% (w/v) DDM to remove free LptDE. The column was washed with 20 column volumes of wash buffer and bound LptDE–RBP complex and RBP were eluted with 3 column volumes of elution buffer + 0.15% (w/v) DDM. The eluate was concentrated and further purified by SEC using a Superdex 200 Increase 10/300 GL column equilibrated in 10 mM HEPES, pH7.5, 100 mM NaCl, 0.04% (w/v) DDM.

### Peptide Identification in LptDE–RBP_Oeko_.

A 10 ug sample of LptDE–RBP_Oeko_ from collection 2 was analyzed without enzymatic digestion using an 8 cm Performance C18 column with a 100-step gradient. Data were acquired on a Bruker timsTOF HT operated in PASEF-DDA mode. Spectra were searched with FragPipe against the *E. coli* UniProt subset, supplemented with the RBP_Oeko_ sequence and common contaminants, using peptidomic parameters without enzyme specificity. Results were filtered at a 1% FDR. A blank injection (0.1% TFA) was run immediately before sample acquisition and no peptide matches were detected.

### CryoEM Sample Preparation, Data Collection, and Data Processing.

#### Data collection.

Quantifoil R1.2/1.3 Cu 300 mesh holey carbon grids were glow-discharged in air using a PELCO easyGlow system (12 mA, 30 s). Samples were applied to the grids (3 μL), and blotting followed by plunge freezing into liquid ethane was performed using a Vitrobot Mark IV (Thermo Fisher Scientific) operated at 4 °C and 100% humidity. Grids were blotted for 6, 8, and 10 s with a blot force of six. Cryo-EM movies were collected on a Titan Krios microscope operating at 300 kV. Detailed data acquisition parameters are provided in *SI Appendix*, Table S1.

#### Data processing and structure refinement.

Movies were imported into CryoSPARC (49) for processing. Motion correction and CTF estimation were performed with micrographs with poor CTF fits or low image quality excluded. Particles were extracted with Fourier cropping to reduce data size, followed by one or two rounds of 2D (two dimensional) classification. Ab-initio reconstruction was conducted using both selected “good” 2D classes and excluded particles (junk). One to three rounds of heterogeneous refinement were performed using both the protein and junk classes. Particles from the final protein class/es were re-extracted at full resolution and final reconstructions were obtained using nonuniform (50) and local refinement. Detailed processing workflows for each structure are provided in *SI Appendix*, Figs. S1, S6, S11, and S15. Structural models were generated using AlphaFold3 (38) and fit into cryo-EM density maps using UCSF ChimeraX (51). Rigid-body refinement was then performed in PHENIX (52). Models were then manually rebuilt and corrected in Coot (53). Final real-space refinement was carried out in PHENIX.

### Phage Spot Assays.

*E. coli* strains (Bl21(DE3)*Δcyo*, *imp4213*, *R2*, *R3,* and *R4*) were grown in 5 mL LB at 37 °C with shaking. LB soft agar (0.5% w/v) was prepared and melted prior to use. The soft agar was supplemented with 20 mM MgSO_4_ and 5 mM CaCl_2_ and inoculated with the appropriate bacterial strain to an OD_600_ of ~0.1, poured onto LB agar plates, and allowed to solidify at room temperature. The Oekolampad phage (bas18) was freshly diluted to 10^−2^ to 10^−6^ in 20 mM HEPES (pH 7.5), 10 mM MgCl_2_, and 10% glycerol. Once the top agar had set, 5 μL of each phage dilution was spotted on the surface. Plates were allowed to absorb the spots and were then incubated at 37 °C for 24 h.

### SDS/EDTA Sensitivity Assays.

*E. coli* strains *R2*, *R3*, *R4*, Bl21(DE3)*Δcyo*, and *imp4213* were compared for sensitivity to detergent and chelator stress. LB agar plates were prepared with 0.5% SDS and 0.5 mM EDTA. Overnight cultures of each strain were used to inoculate 5 mL of fresh LB and grown at 37 °C with shaking until reaching an OD_600_ of 0.4 to 0.6. Cultures were then diluted to an OD_600_ of 0.1 in LB. Ten-fold serial dilutions were prepared in LB, and 1 μL of each dilution was spotted onto the SDS-EDTA plates. Plates were incubated at 37 °C, and growth was assessed after 16 to 24 h to evaluate detergent and chelator sensitivity across strains.

### Molecular Dynamics Simulations.

The RBP structure predicted by AF3 (38) was taken to model the missing region from the cryo-EM density. The AF3 RBP model and the cryo-EM structure of the RBP were aligned using PyMOL (54), which gave an rmsd of 0.581 Å for 223 Cα pairs out of 257. The structure was then further aligned on five residues either preceding or succeeding the missing region (residues 26, 112, 143, 205, and 235), giving an rmsd of 0.262 Å. Residues 1 to 25, 113 to 142, and 206 to 234 of the RBP were modeled using the aligned AF3 model. This region was energy minimized while the rest of the protein complex’s atoms were restrained using position restraints of 10,000 kJ/mol.

Each simulation system had three replicates, and each replicate was individually prepared using the CHARMM-GUI membrane builder (55–57). The protein complex was embedded within a model *E. coli* outer membrane, containing *E. coli* R1-LPS without O-antigen in the outer leaflet, and 1-palmitoyl 2-cis-vaccenic phosphatidylethanolamine, 1-palmitoyl 2-cis-vaccenic phosphatidylglycerol, and cardiolipin (1-palmitoyl 2-cis-vaccenic 3-palmitoyl 4-cis-vaccenic diphosphatidylglycerol) in the inner leaflet in a 90:5:5 ratio. The system was solvated with water and K^+^ Cl^−^ ions at 0.2 M. Calcium ions were used to neutralize negatively charged phosphate groups of LPS. Each simulation box was 14 nm × 14 nm × 16 nm.

Energy minimization of the systems was conducted using the steepest descent algorithm until the maximum force was below 500 kJ/mol. Systems were first equilibrated within the NVT ensemble for 200 ps, with a time step of 1 fs, using the v-rescale thermostat (58), with a time constant of 1 ps, at a temperature of 313 K. Initial velocities were assigned randomly for each replicate. Subsequent further equilibration and production runs were conducted within the NPT ensemble using a time step of 2 fs. Pressure was maintained at 1 bar using the c-rescale barostat (59) with a time constant of 5 ps. Covalent bonds involving hydrogen were constrained using the P-LINCS algorithm (60). Electrostatic interactions were described using the Particle Mesh Ewald method (61) Equilibration simulations of duration 31 ns of were performed while gradually releasing position restraints on amino acids and lipids. A further 30 ns of equilibration was performed without any position restraints. It was within this state that the removal of the bound S0 strand resulted in a very rapid closing of the lateral gate. Production runs were 2 µs. All simulations used the CHARMM36m (62) forcefield and MD simulations were performed using the GROMACS (63) software package (version 2022.4).

HOLE (39, 64) was run using the HOLE2 implementation within the MDAnalysis (65, 66). The center of mass between Asn345 and Ile777 served as the starting point for HOLE analysis in both the closed and open system. The DSSP algorithm (67) was used to analyze secondary structure, implemented through MDTraj (68). Hydrogen bond numbers were calculated using MDAnalysis, using the backbone atoms of the S0 peptide and the surrounding beta barrel. A quite permissive Donor-H-Acceptor angle cutoff for hydrogen bonds of 135.0°, distance cutoff between donor–hydrogen pairs of 2.5 Å, and distance cutoff between donor and acceptor of 3.5 Å was used, although this falls well within the Baker-Hubbard definition (69).

### Minimum Inhibitory Concentration Assays (MICs).

MICs were carried out according to the Clinical Laboratory Standards Institute (CLSI) M100 guidelines. Bacterial cell strains were spread onto LB-agar plates and grown overnight. For *E. coli* strains: ATCC25922, *imp4213*, *ΔsurA*, *ΔwaaD*, *ΔdsbA*, *ΔdsbC*, *ΔlptM,* and DC2, inoculate was prepared by suspending colonies from the plate into 0.9% saline to a cellular density corresponding to a 0.5% McFarland standard, then diluted 1:400 into either Mueller-Hinton broth (MHB, 70192) (Millipore). For hyperporinated *E. coli* strain GKCW102 and its control parent strain GKCW101, an overnight starter culture was grown in MHB then used to inoculate fresh media and grown to an OD_600_ of 0.3. FhuA pore expression was induced by the addition of arabinose to a final concentration of 0.1%, and cells were grown to an OD of 1. This culture was diluted 1:2,000 and used as the MIC inoculate. 200 µL of inoculum from each strain was added to a 96-well plate containing a dilution gradient (with a top concentration of 64 µg/mL) of S0 peptide solubilized in DMSO. Both the L- and the D- form of the peptide were assayed in the same way. All cell lines were separately treated with antibiotic controls. Plates were incubated overnight at 37 °C, then checked for growth following CLSI guidelines.

## Supplementary Material

Appendix 01 (PDF)

Dataset S01 (XLSX)

Movie S1. The LptDEM structure. Map and cartoon showing bound LptM (green) within the LptD lumen.

Movie S2. LptDE with bound RBPOeko, emphasising the connection between LptD strands S0 and S1 (red). Density from dataset 2 is shown for the connecting segment.

Movie S3. LptDE with bound RBP_Oeko_, emphasising the difference between the closed and open states of LptD. The movie starts with a density map (dataset 2) followed by a cartoon. Following the top view, RBP (magenta) is removed, and the cartoon is morphed into the closed state, with the S0 segment (red) occupying its position within the jellyroll domain. A surface view of the closed state follows. Subsequently, the closed state morphs back into the open state and ends with a surface view (RBP not shown for clarity).

Movie S4. Rtp45 binding to LptDEM. Movie starts with a cartoon of LptDE from the LptDEM dataset (LptD in tan, LptE in blue). The barrel is clipped open and morphed into the LptDE conformation from the LptDEM-Rtp45 complex and Rtp45 (maroon) moves into position. A surface representation of RBP (magenta), from the SfLptDE-RBP_Oeko_ complex is displayed to illustrate clashes with EL4 (highlighted in green). The map from the LptDEM-Rtp45 dataset is then shown. LptM is hidden for clarity.

## Data Availability

Original data created for the study are or will be available in a persistent repository upon publication. CryoEM maps have been deposited in the Electron Microscopy Data Bank and atomic coordinates have been deposited in the Protein Data Bank with accession codes EMD-54169/9RPR (LptDEM) (70, 71), EMD-54171/9RPT (SfLptDE–RBP monomer dataset 1) (72, 73), EMD-54173/9RPW (SfLptDE–RBP dimer) (74, 75), EMD-54175/9RQI (SfLptDE–RBP monomer dataset 2) (76, 77), and EMD-54170/9RPS (SfLptDEM-RTP45) (78, 79).
