# Supplementary material for: Small siphophage binding to an open state of the LptDE outer membrane lipopolysaccharide translocon
Source: Proc Natl Acad Sci U S A. 2025 Nov 26;122(48):e2516650122. doi: 10.1073/pnas.2516650122 (PMC12685063; doi:10.1073/pnas.2516650122)
Supplement: Supplementary file 1 — Appendix 01 (PDF) [file pnas.2516650122.sapp.pdf]

Supplementary Information

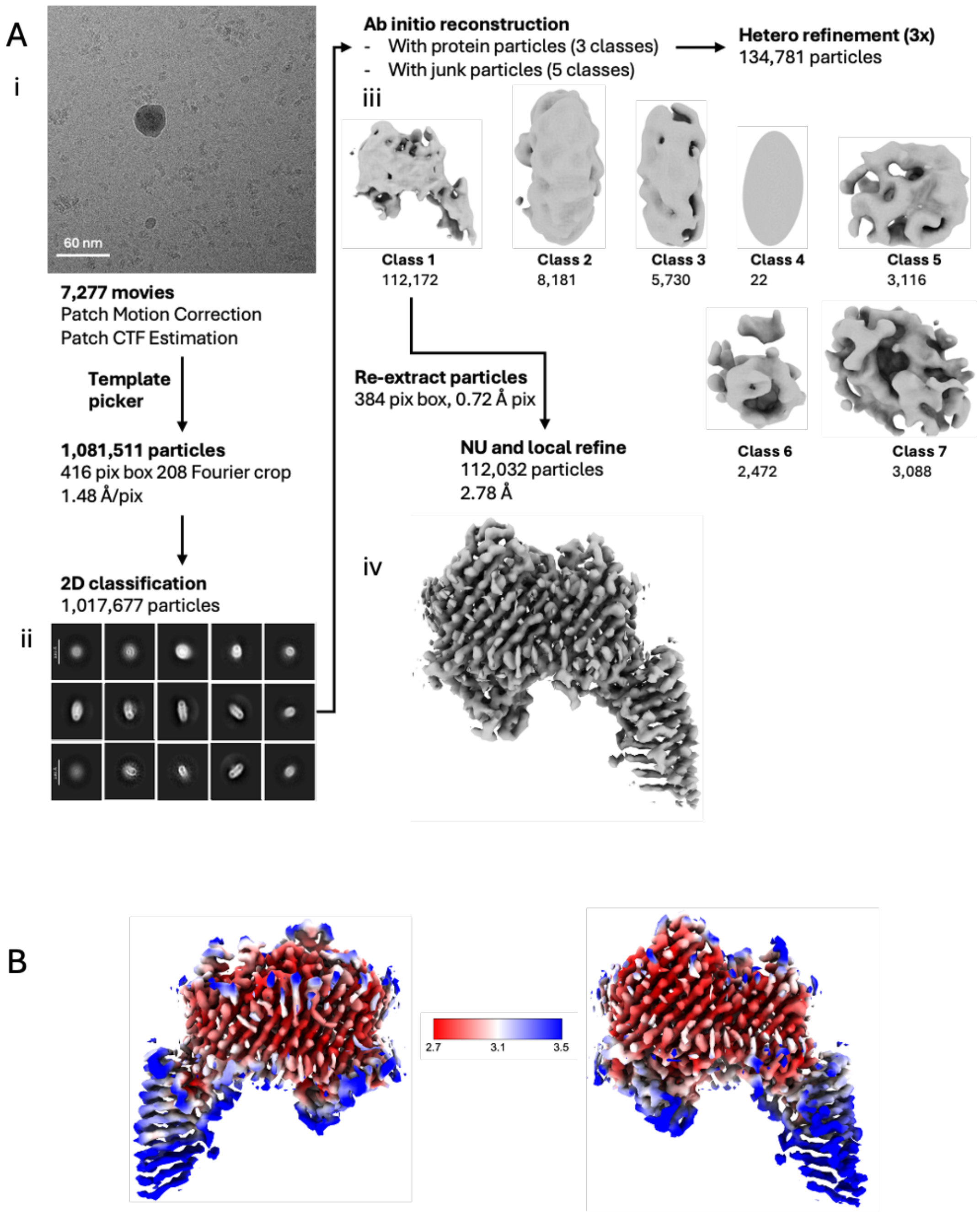

Fig. S1 Cryo-EM data processing for *Sf*LptE-*Ec*LptD-*Ec*LptM complex. (A) Workflow summary. 7,277 movies were collected and imported into CryoSPARC (1). After motion correction and CTF estimation, low-quality micrographs were excluded. Particles were extracted with Fourier cropping and subjected to 2D classification to remove obvious junk (i, example micrograph; ii, representative 2D classes). Ab-initio reconstruction produced volumes used in three rounds of heterogenous refinement with one "good" and six "junk" classes (iii, volumes from final round). particles were re-extracted at full resolution for non-uniform (2) and local refinement, yielding a final 2.78 Å map (iv, final volume). (B) Local resolution estimation of the final map.

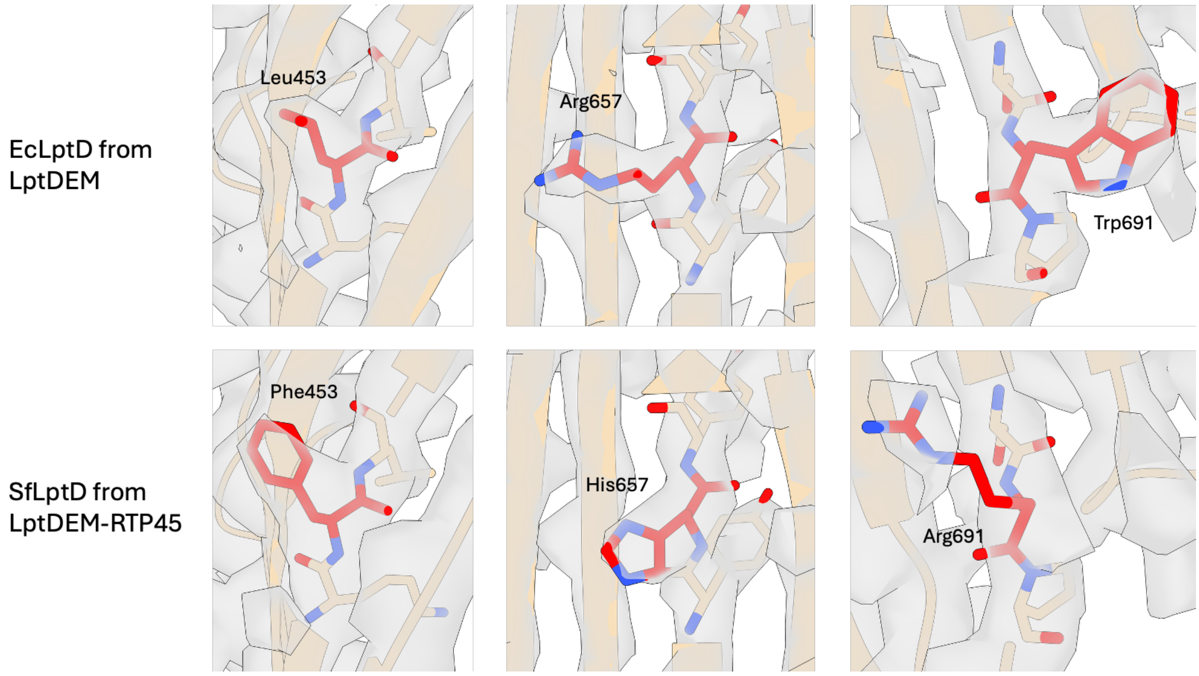

Fig. S2 Amino acid differences between *E. coli* and *S. flexneri* LptD. The three amino acid differences at positions 453, 657 and 691 are highlighted in red. These are shown in the *Ec*LptD-*Sf*LptE-*Ec*LptM and the *Sf*LptDE-*Ec*LptM-RTP45 maps and models. The single different amino acid in the LptE C-terminus (residue 190) is not visible in any map.

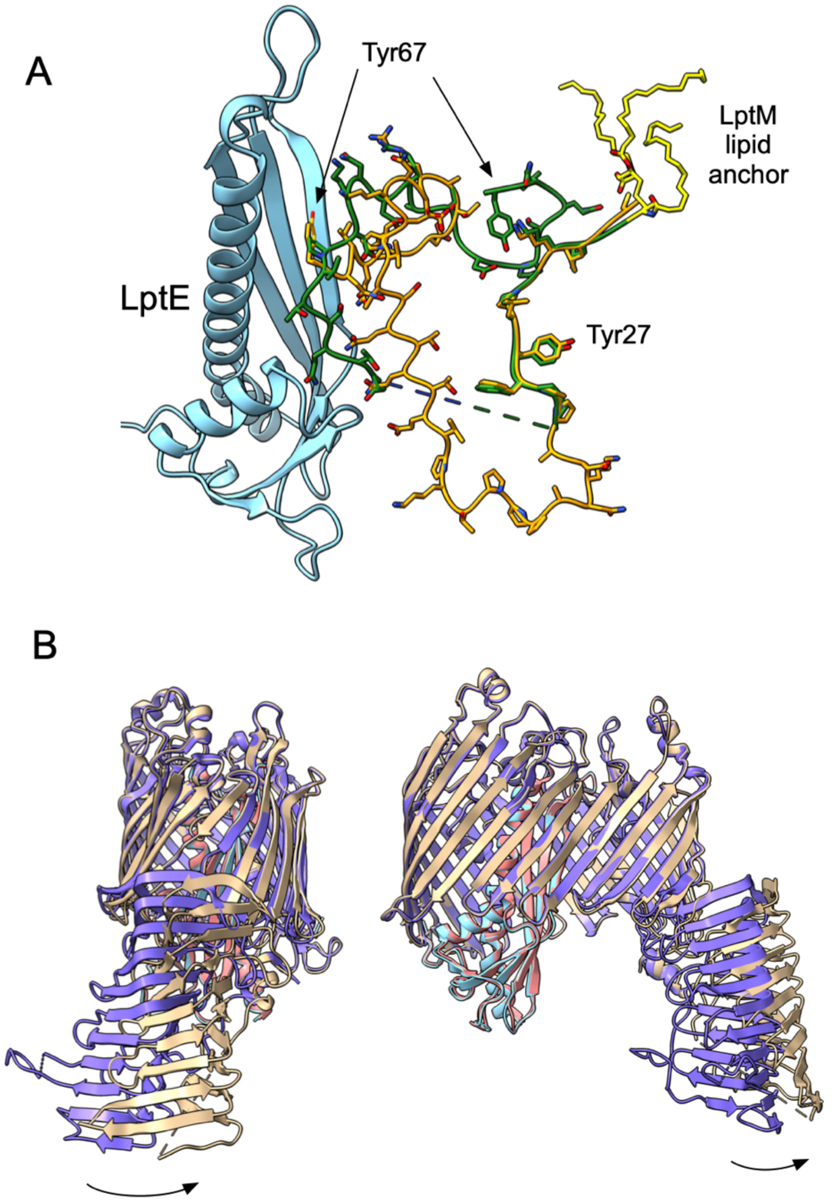

Fig. S3 Structures compared to AF3 predictions. (A) LptM within the LptDE structure (green) and the predicted AF3 model in orange (Yang 2023). For reference, LptE is shown as well. Structures were superposed on LptD; apart from LptM, the structural differences are negligible. The arrows point to the C-terminal Tyr67 LptM residues, highlighting the large differences between the experimental LptM structure and the AF3 model. (B) Superposition (on LptD) of the cryo-EM structure (coloured as in Fig. 1) with the X-ray crystal structure of SfLptDE, with LptD coloured purple and LptE pink (PDB 4Q35; Dong 2014). The arrows highlight the different positions of the N-terminal jellyroll domains. LptM, present only in the cryo-EM structure, is not shown for clarity. Compared to the X-ray crystal structure (PDB 4Q35), the LptDE cryo-EM structure is largely identical except for the N-terminal jellyroll domain. In the cryo-EM structure and the identical AF3 model (not shown for clarity), the

jellyroll domain has moved/rotated approximately as a rigid body, breaking interactions between the barrel and the jellyroll domain (3). In the crystal structure, the different LptDE monomers pack via end-to-end stacking of the jellyroll domains, and it is possible that this has induced a different jellyroll domain position in the crystal. Given our data suggest LptDE purifies with tightly bound LptM, this suggests that the crystallisation process has selected for apo-LptDE. Interestingly, a crystal structure for *Pseudomonas aeruginosa* LptDE (expressed in *E. coli*) was recently published (PDB ID 8H1R; (4)) in which density for *E. coli* LptM residues Cys20-Asp32 is visible at the same location as in our cryo-EM structure. In addition, the recent cryo-EM maps of NgLptDE (5) also contains density consistent with bound LptM, although it was not modelled.

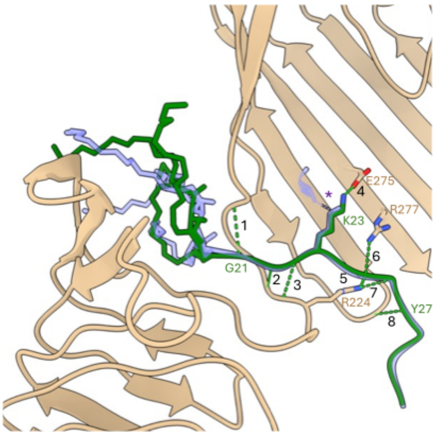

		1	2	3	4	5	6	7	8
	Residue	GLY	LEU	LEU	LYS	GLY	GLY	PRO	TYR
	No.	21	22	22	23	24	24	25	27
LptM	Atom	N	N	O	NZ	O	O	O	N
	Residue	PHE	ARG	ARG	GLU	ARG	ARG	ARG	PRO
	No.	228	225	225	275	224	277	224	253
LptD	Atom	O	O	N	OE1	NH1	NH2	NH1	O

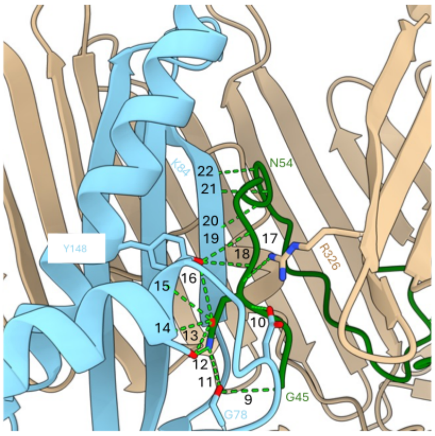

		9	10	11	12	13	14	15	16	17	18	19	20	21	22
	Residue	GLN	GLN	GLN	GLN	GLN	GLN	GLN	GLN	GLN	SER	SER	ASP	ASP	ASN
	No.	45	47	47	47	47	47	47	47	47	48	48	52	52	54
LptM	Atom	N	N	NE2	NE2	OE1	OE1	OE1	OE1	O	N	O	N	O	N
	Residue									ARG					
	No.									326					
LptD	Atom									NH2					
	Residue	GLY	ASP	SER	SER	SER	SER	LEU	PRO		TYR	TYR	ILE	LYS	LYS
	No.	78	42	40	48	48	48	47	46		148	148	82	84	84
LptE	Atom	O	OD2	OG	OG	OG	N	N	N		OH	OH	O	N	O

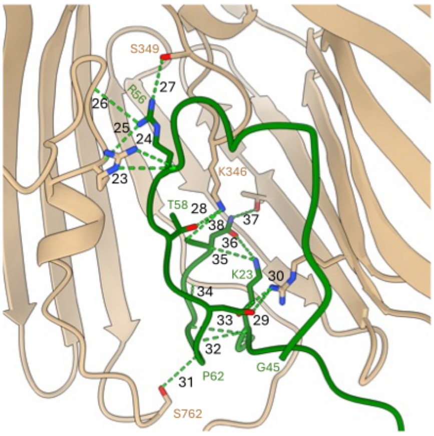

		23	24	25	26	27	28	29	30	31	32	33	34	35	36	37	38
	Residue	ASP	ASP	ARG	ARG	ARG	THR	ASP	ASP	PRO	SER	SER	GLN	ASN	ASN	ASN	TYR
	No.	55	55	56	56	56	58	60	60	62	63	63	64	66	66	66	67
LptM	Atom	O	O	NH1	NH1	NH2	OG1	OD2	OD1	O	O	N	O	O	OD1	ND2	O
	Residue	ARG	ARG	ARG	ASN	SER	LYS	ARG	ARG	SER			ILE			THR	LYS
	No.	774	774	774	776	349	346	277	277	762			230			260	346
LptD	Atom	NE	NH2	O	O	OG	NZ	NH1	NH1	OG			N			OG1	NZ
	Residue										LYS	LYS		LYS	LYS		
	No.										23	23		23	23		
LptM	Atom										N	O		NZ	NZ		

Fig. S4 Polar interactions between EcLptM and LptDE. Different orientations are shown with the interactions indicated in the tables next to the panels. LptM from RTP45-LptDEM is overlaid and coloured lilac in the top panel. The asterisk in the top panel indicates the LptM K23 to LptD T258 hydrogen bond that is present in the RTP45-LptDEM structure but not in LptDEM. Backbone atoms are not shown for clarity.

A

```
Rtp44       MAQGIYIDLNDGRKPMTITSGMRGLSFAGNFNVSSGGFT---KTFPISGSN-GSSKFLLP   56
OekoRBP     MGAGILIDYNDGRPRMEITAGLRAPSYCTSFNQRAQSNKTLTINTPLTAGSQVVVALTRP   60
            *. ** ** ****  * **:*:*. *:. .**  : . .    . *::...     :  *

Rtp44       RNGAYSFELDRG-VEIYYINGFSATNTQGTITIGGENAYN--DRITQFSGTVVEVLRA-S   112
OekoRBP     VEVIEVFDQTLVIPDPFYVTSVT-RNGNSGITLRGDDAYGAYSGLPQWAGVIMEVLPVGS   119
             :    *:      : :*:...:  * :. **: *::**.  . : *::*.::*** . *

Rtp44       TGQGIYVADSTDFASITTRDRLLTCKFSGTVSFTNSYTMPIAGIPFGKWNNSGVSVEFDG   172
OekoRBP     RNAGLLVANSTDFTAISNVAKLMTCRYAKRVRVNGSMALPVSGVPFARWDDGNVSVGFDG   179
             . *: **:****::*:.  :*:**::  * ...* ::*::*:**.:*::..*** ***

Rtp44       NRTLYCTNINTKDTGNTPGSVTLDLIIFQQVAPTPGPGINIFNSNNQCTFSTLTRPLVVS   232
OekoRBP     GSIIVR-NASYGGIDDVAASVDMDLVIFNNTPPTPGTGITMTNNQNQVTFSTVNKPFVYD   238
            .  :   * .   . .:. .** :**:**::. **** **.: *.:** ****:.:*:* .

Rtp44       GFVTLTSSNQSIGNSYFPILRCGFNTRAIPAYKELRNKGVVMTGGNVRSGTGTRIVRYGI   292
OekoRBP     RTINIGTSDQNIGNSLIQLSYTGALIQNNGGYNHVRMNGIRMAGNNVRVAKNRVIGNYSR   298
              :.: :*:*.**** : :   *   :   .*:.:* :*: *:*.*** ...   * .*.

Rtp44       GN----RPDVTLGIPLPYLPDMY      311
OekoRBP     QQFQMPGKNIAVPTPLLVIPNMY      321
             :      ::::  **  :*:**
```

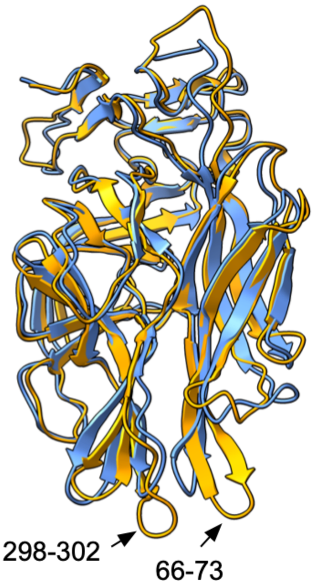

B

```
Rtp45       MTNLIKLSIIAAAAIMMVGCSSGPRPDGWCATQASGVCVAKWKNGVVVPAGEVDVRYDGI    60
OekoSE      -----MKTLIMICAVLLSGCAV-ERNNGDCITVAYGSCMLRYVDGQKVPAGDVDMRFTGL    54
                   ::*  .*::: **:   * :* * * * * *: :: :*  ****:**:*: *:

Rtp45       KGSSGS----VRDYGSKEW   75
OekoSE      SSDSDGNFSGTVSVKSREW   73
            ...*..    . .  *:**
```

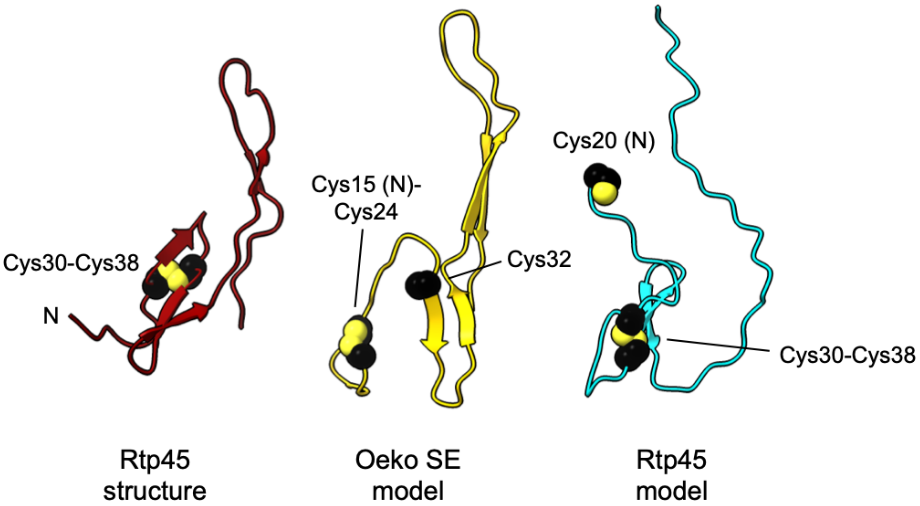

Fig. S5 Structural comparisons of LptD-targeting RBPs and SE proteins. (A) Sequence alignment of Rtp44 (cornflower blue) and RBP_{Oeko} (orange), with a superposition of AF3-predicted models on the right. The divergent tips of two loops that likely mediate slightly different interactions with LptD are underlined in the alignment and indicated by arrows in the cartoon. (B) Sequence alignment of Rtp45 and SE_{Oeko}. The lipid anchor cysteine is indicated in cyan, and the cysteines forming the intramolecular disulphide in yellow. The bottom panel shows (from left) the experimental Rtp45 structure and representative AF3 models of SE_{Oeko} and Rtp45.

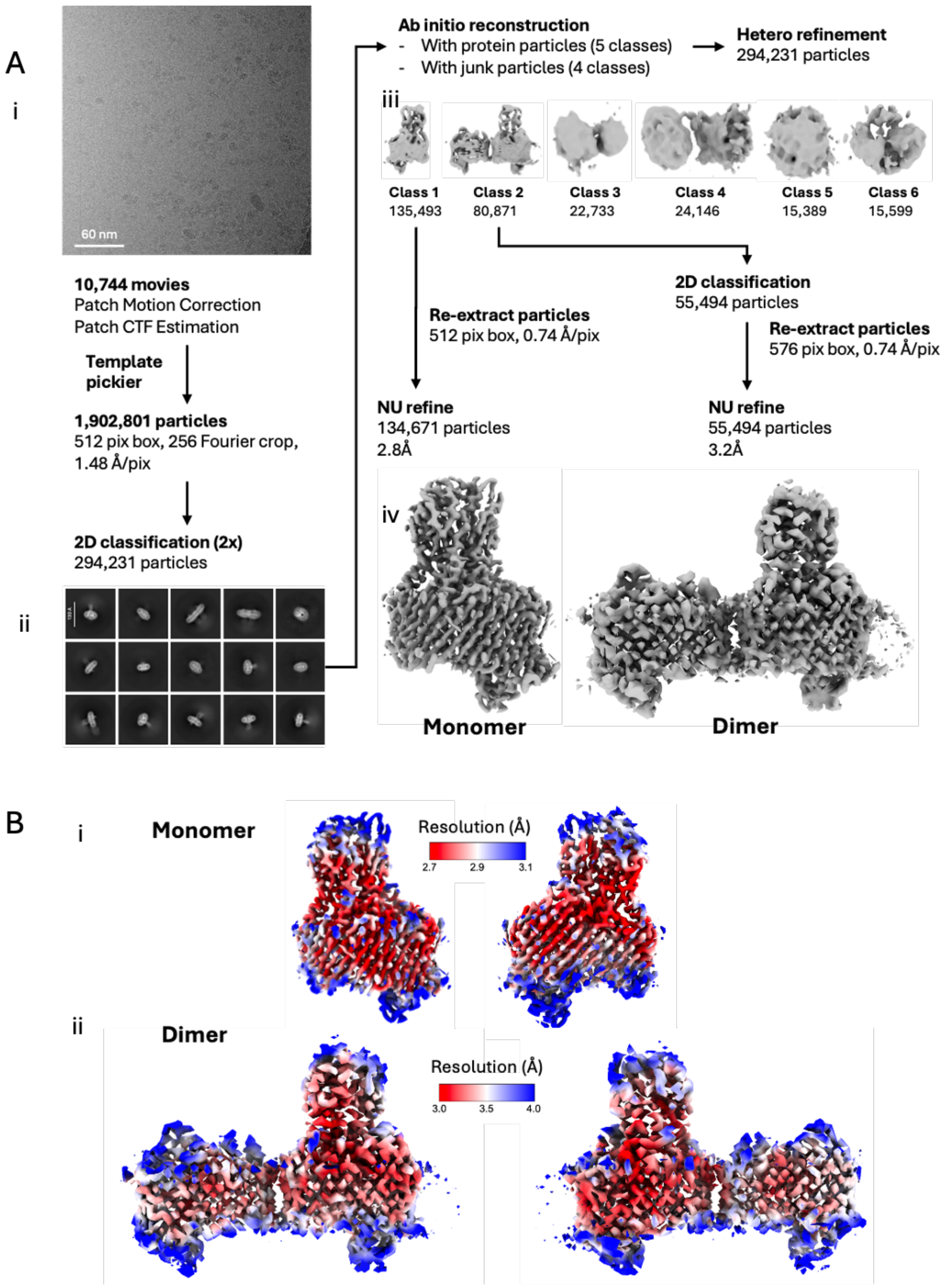

Fig. S6 Cryo-EM data processing for the *Sf*LptD-RBP dataset 1 (monomer and dimer). (A) Workflow summary. 10,744 movies were collected and process in CryoSPARC. After motion correction and CTF estimation, low-quality micrographs were removed. Particles were extracted with Fourier cropping and subjected to two rounds of 2D classification (i, example micrograph; ii, representative 2D classes). Ab-initio reconstruction followed by heterogenous refinement using one monomer, one dimer and four junk classes (iii, volumes). Monomer and dimer particles were re-extracted at full resolution for non-uniform refinement, producing final maps of 2.8Å (monomer) and 3.1Å (dimer) (iv, final volumes). (B) Local resolution estimation of final maps: (i) monomer, (ii) dimer.

A

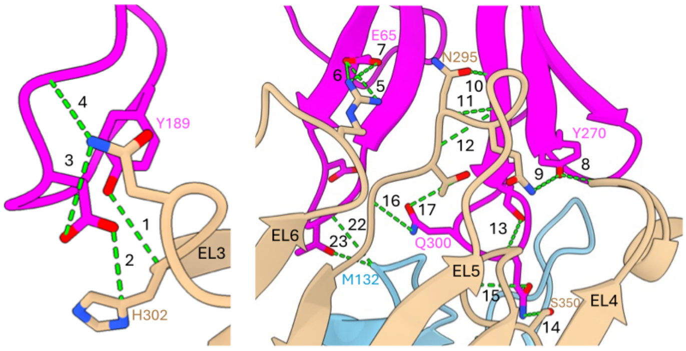

		1	2	3	4	5	6	7	8	9	10	11	12	13	14	15	16	17	18	19	20	21		22	23
RBP	Residue	TYR	ASP	ASP	ASP	GLU	GLU	GLU	TYR	TYR	ASN	ASN	ASN	SER	GLN	GLN	GLN	GLN	ASP	TYR	GLN	GLN	RBP	THR	THR
	No.	189	193	193	194	65	65	65	270	270	295	295	295	297	299	299	300	300	68	98	302	302		70	70
	Atom	OH	OD1	OD1	O	OE1	OE1	OE2	OH	OH	N	O	O	OG	NE2	OE1	NE2	OE1	OD2	O	O	N		O	OG1
LptD	Residue	GLU	HIS	ASN	ASN	ARG	ARG	ARG	PRO	GLN	ASN	ASN	THR	GLY	SER	ASP	SER	THR	LYS	THR	TYR	TYR	LptE	MET	MET
	No.	301	302	304	304	421	421	421	337	383	384	384	385	353	350	352	386	385	670	674	671	671		132	132
	Atom	0	ND1	ND2	ND2	NH1	NH2	NH2	O	NE2	OD1	N	N	N	OG	N	O	OG1	N	OG1	OH	OH		N	N

B

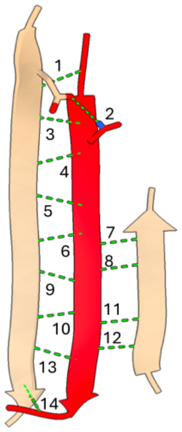

	S0			LptD		
	Residue	Number	Atom	Residue	Number	Atom
1	ILE	198	O	ASN	750	N
2	ASN	200	ND2	ASP	749	OD1
3	ASN	200	N	ASN	750	O
4	ASN	200	O	ILE	752	N
5	ARG	202	N	ILE	752	O
6	ARG	202	O	PHE	754	N
7	PHE	203	O	TYR	235	N
8	PHE	203	N	TYR	235	O
9	LYS	204	O	PHE	754	O
10	LYS	204	N	ILE	756	N
11	VAL	205	N	ALA	233	O
12	VAL	205	O	ALA	233	N
13	GLY	206	N	ILE	756	O
14	VAL	208	N	VAL	757	O

Fig. S7 Polar interactions between SfLptDE and (A) RBP_{Oeko} or (B) the S0 peptide. Backbone atoms are not shown for clarity.

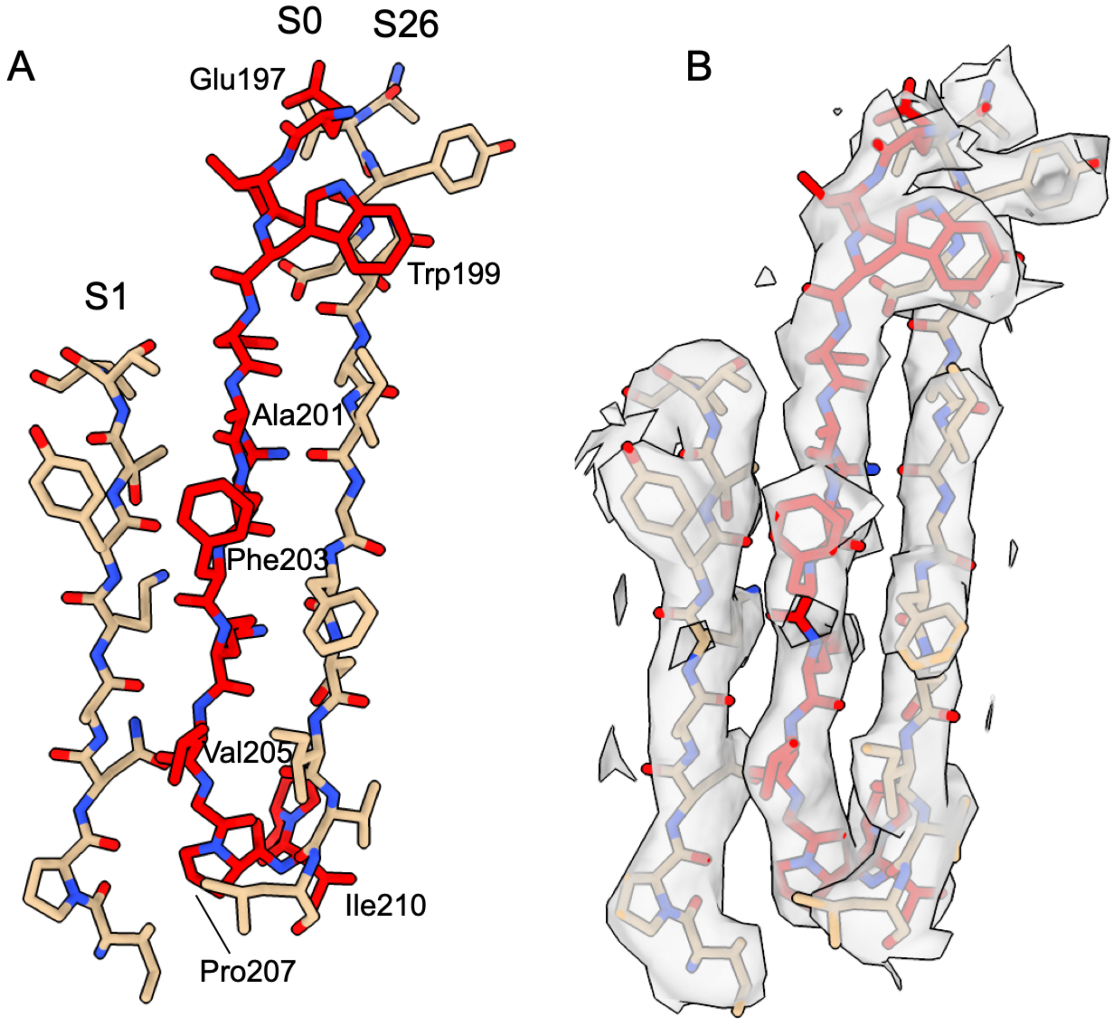

Fig. S8 The lateral gate strand S0 from LptDE-RBP dataset 1. (A) Stick model of LptD strands S0 (red), S1 and S26. Outward facing residues in S0 have been labelled. Corresponding map for dataset 1 of the region in (A).

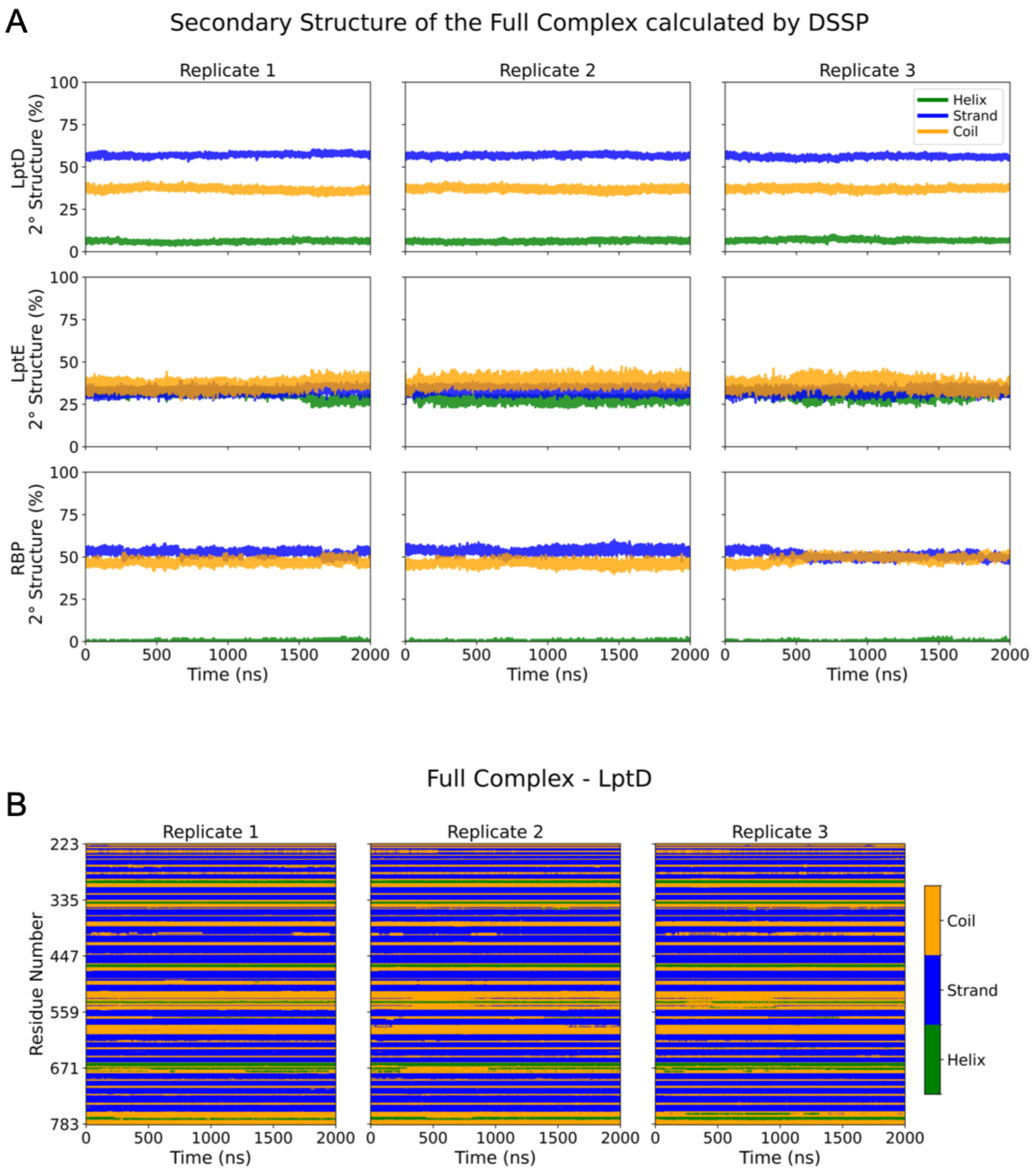
A
Secondary Structure of the Full Complex calculated by DSSP
Replicate 1
Replicate 2
Replicate 3
Helix
Strand
Coil
LptD 2° Structure (%)
LptE 2° Structure (%)
RBP 2° Structure (%)
Time (ns)
B
Full Complex - LptD
Residue Number
223
335
447
559
671
783
Coil
Strand
Helix
Time (ns)

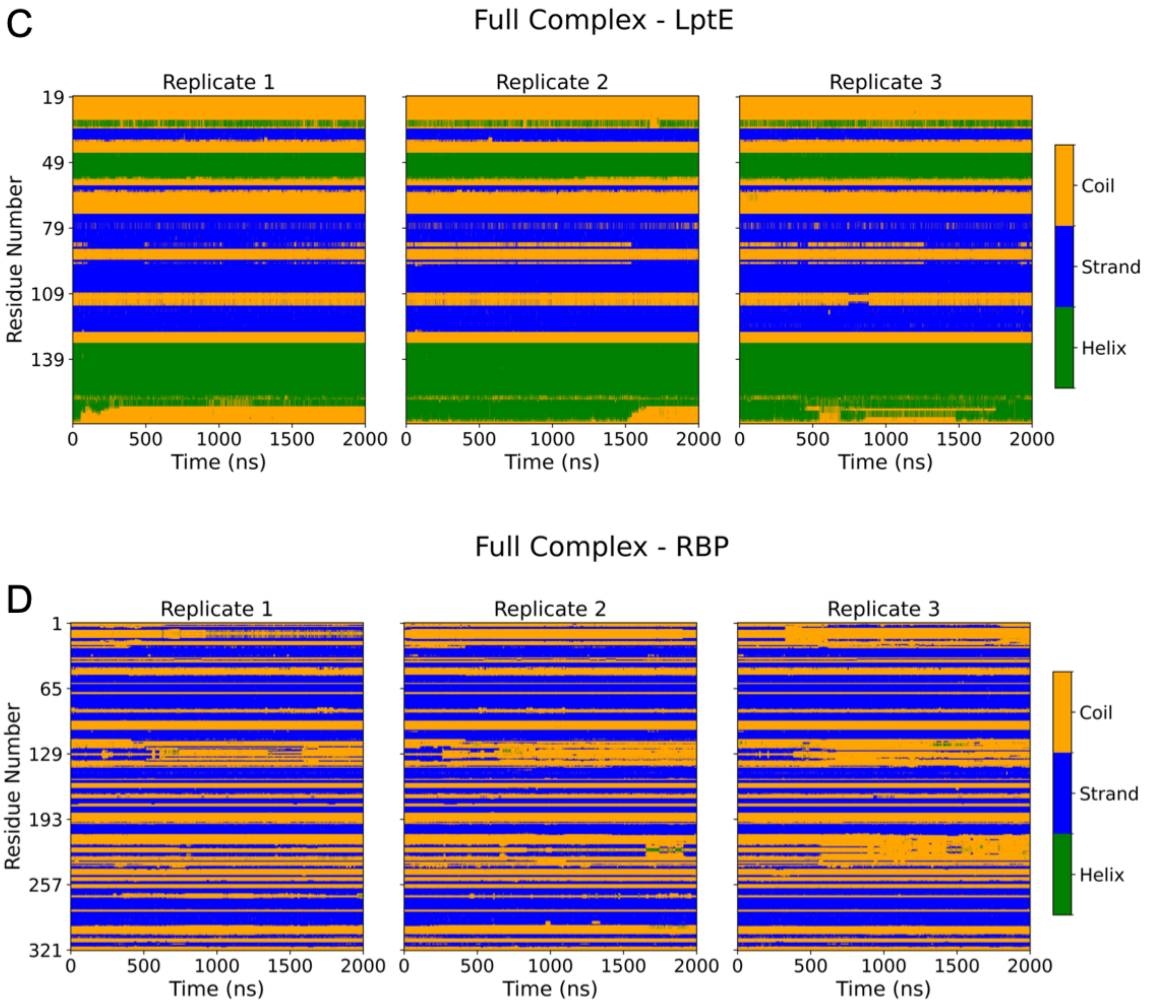

Fig. S9 Stable secondary structure in the SfLptDE-RBP$_{Oeko}$ complex. (A) Molecular dynamics simulations (3 x 2 μs) showing the overall secondary structure for each of the three proteins, as analysed by DSSP. (B-D) Sequence-specific secondary structure for LptD (B), LptE (C) and RBP$_{Oeko}$ (D).

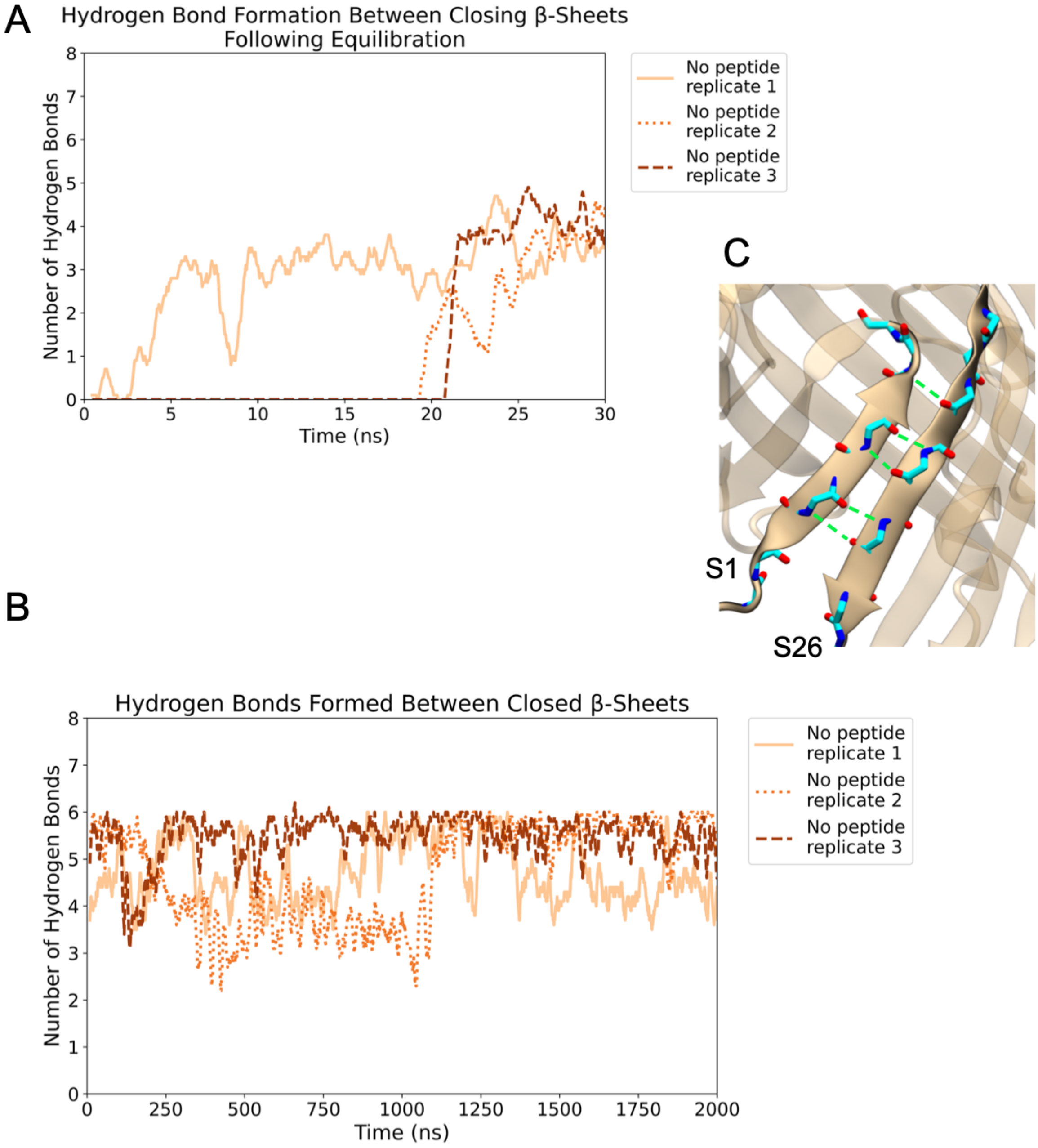

Fig. S10 *In silico* removal of the S0 peptide results in fast and stable lateral gate closure. (A,B) Initial 30 ns and complete trajectories of the three simulations of SfLptDE-RBP$_{Oeko}$ following S0 removal. The traces in (B) are rolling averages over 10 ns. (C) Representative snapshot of the closed lateral gate after S0 removal. Residue side chains are not shown for clarity. Hydrogen bonds are shown as dashed green lines.

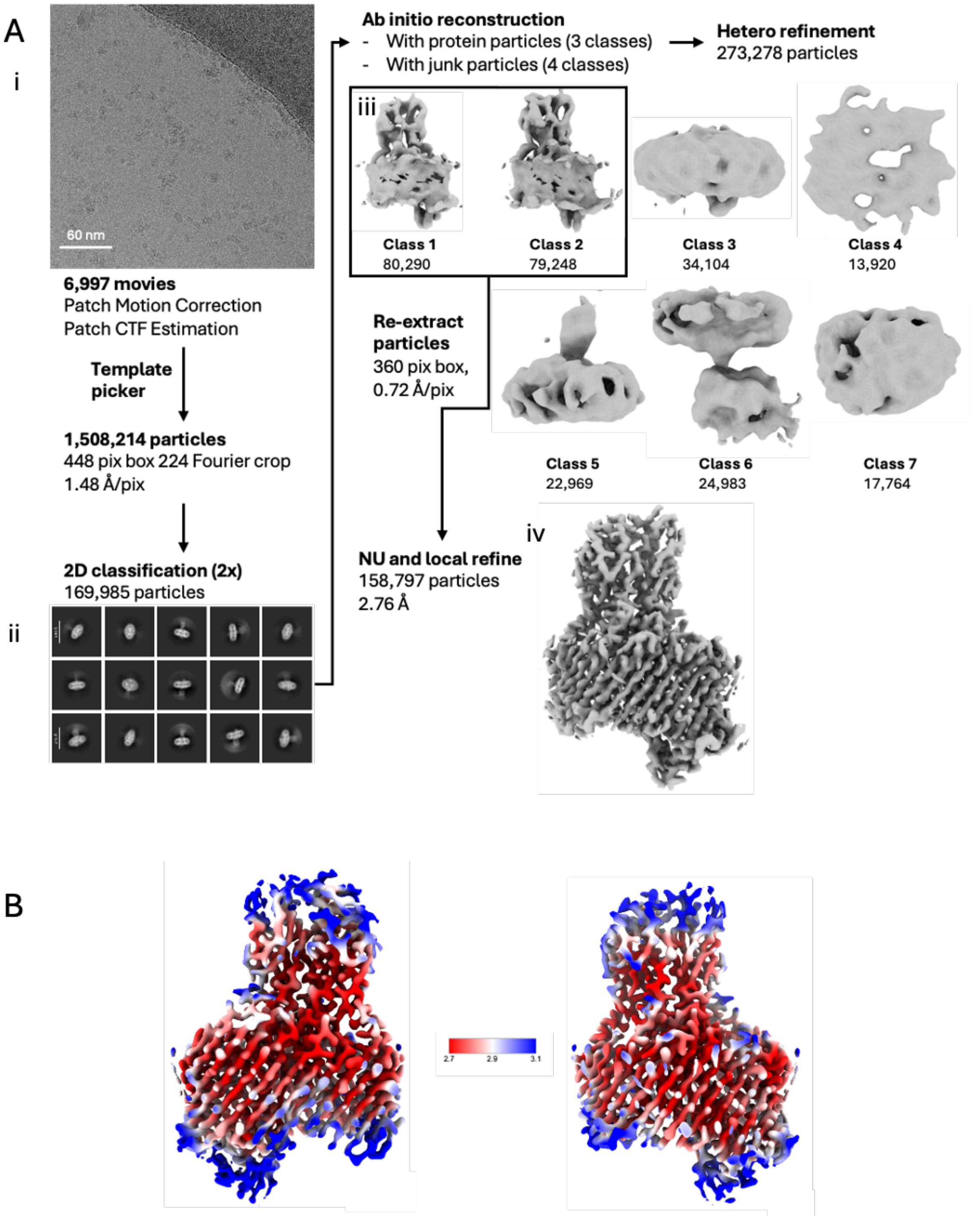

Fig. S11 Cryo-EM data processing for the *Sf*LptD-RBP dataset 2. (A) Workflow summary. 6,997 movies were collected and imported into CryoSPARC. After motion correction and CTF estimation, low-quality micrographs were excluded. Particles were extracted with Fourier cropping and subjected to two rounds of 2D classification (i, example micrograph; ii, representative 2D classes) followed by ab-initio reconstruction and heterogenous refinement using protein and junk volumes (iii, volumes). particles were re-extracted at full resolution for non-uniform and local refinement, yielding a final 2.76Å map (iv, final volume). (B) Local resolution estimation of final map.

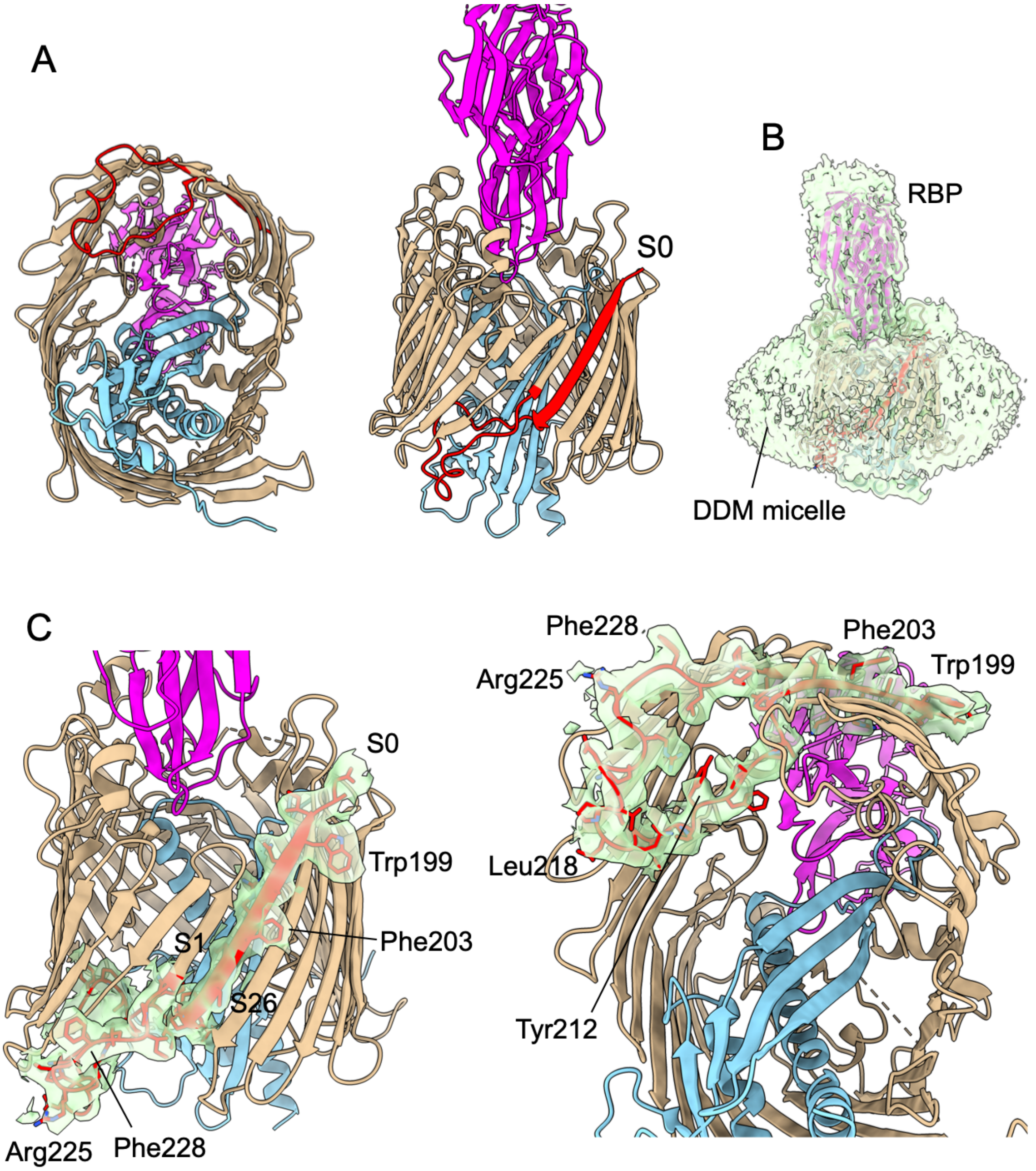

Fig. S12 The lateral gate β-strand S0 is part of the jellyroll domain. (A) Cartoons from the periplasmic space (left) and from the front, showing LptD segment Glu197-Asn232 in red. (B) Low contour map of dataset 2. (C) Cartoons including carved low contour maps (as in B) for the Glu197-Asn232 segment of LptD. See also Movie S2.

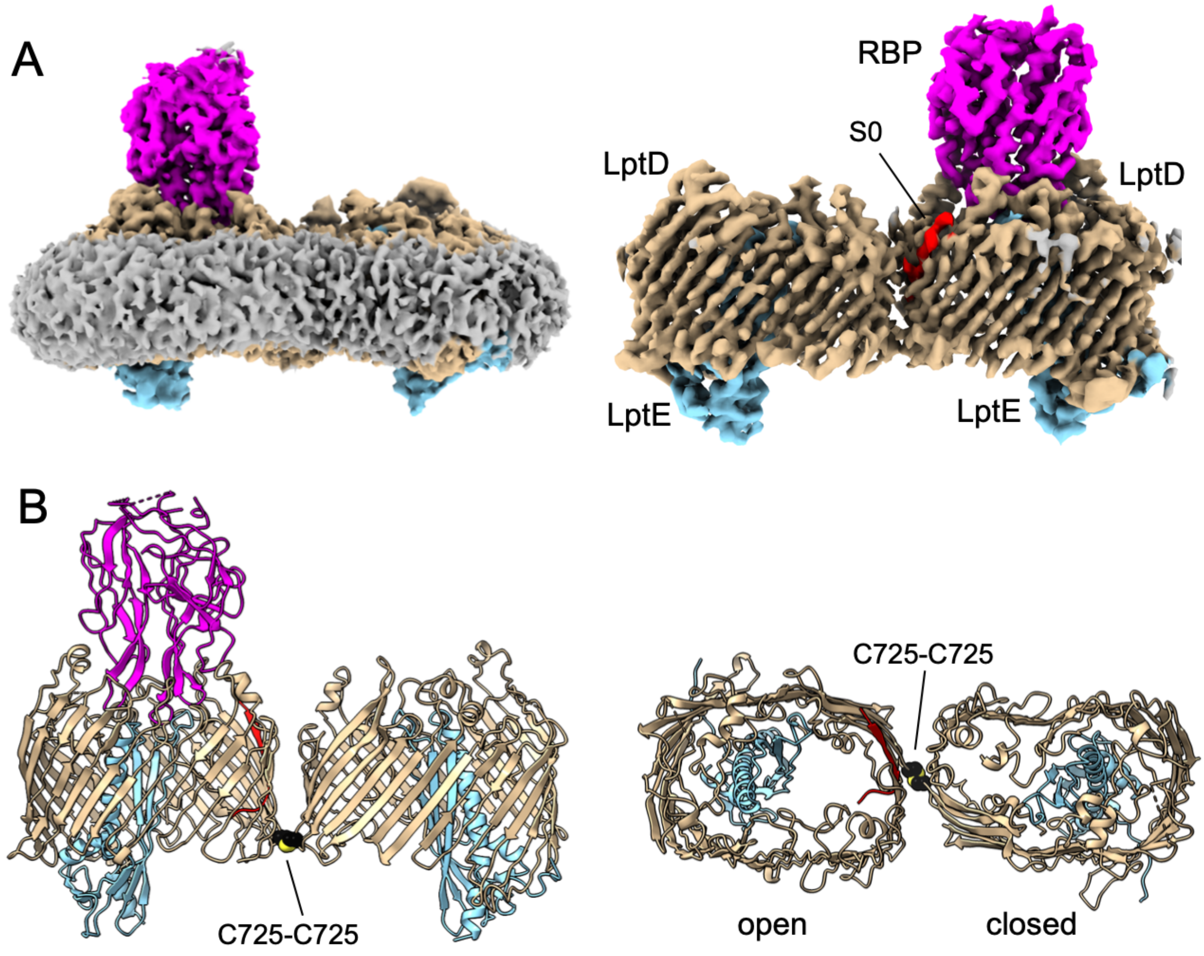

Fig. S13 Structure of dimeric LptDE. (A) Low (left) and high contour maps of LptDE (dataset 1). (B) Cartoons viewed from the OM plane (left) and from the extracellular side. The intermolecular Cys725-Cys725 disulphide bond between LptD protomers is shown as space-filling models (carbons, black; sulphurs yellow). RBP is not shown in the extracellular view for clarity.

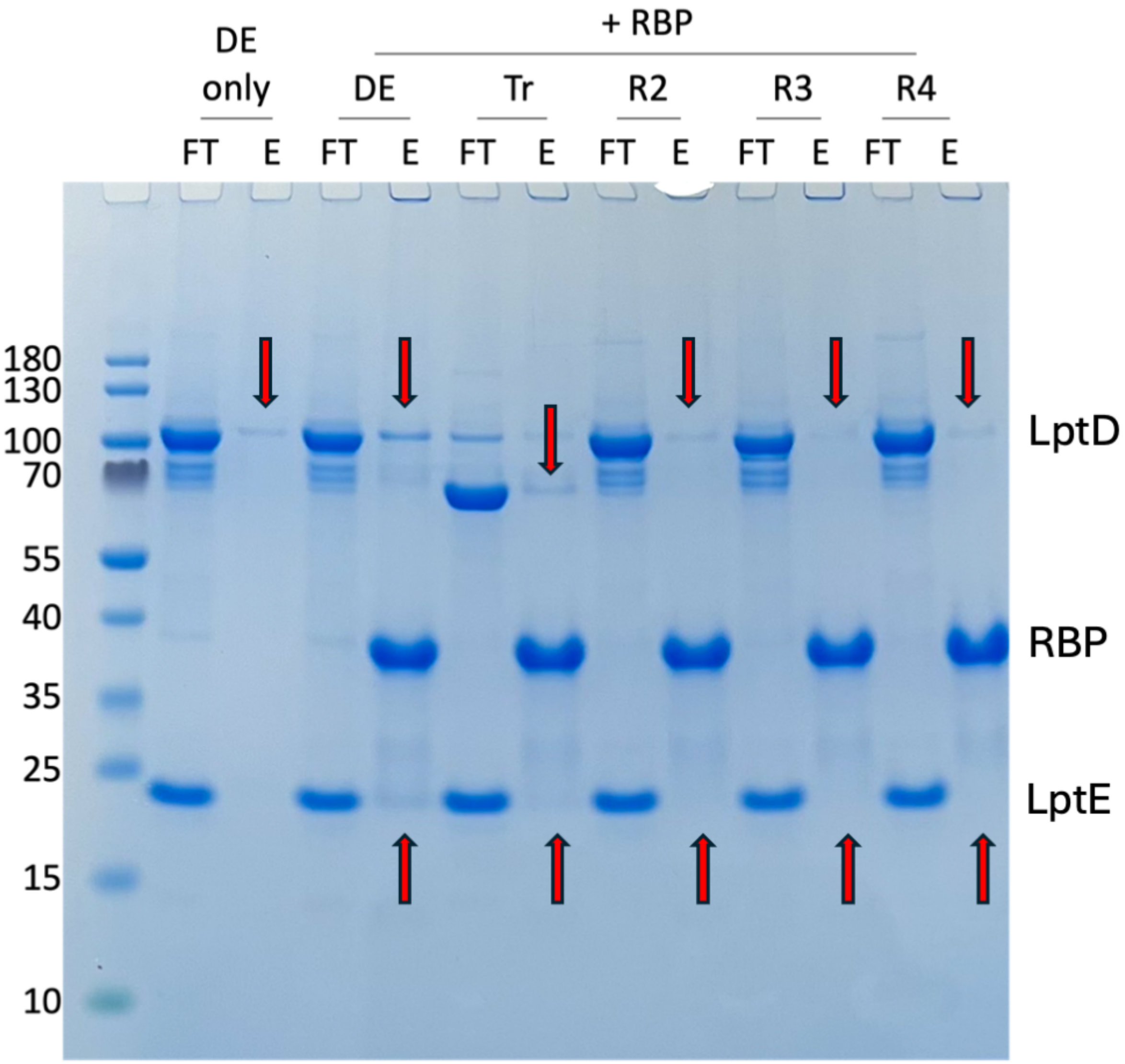

Fig. S14 Pulldown experiments for LptDE +/- RBP incubations. Non-tagged WT and variant LptDE complexes were incubated with 3-fold molar excess of His-tagged RBP for 48 h at 4 C° and subjected to batch IMAC. Only flowthrough (FT) and elution (E) fractions are shown. The arrows indicate the expected positions of LptD and LptE within the elution fractions. DE, wild type LptDE; Tr, truncated LptD without the jellyroll domain (Δ26-201); R2, LptD Y671N; R3, LptD D352Y; R4, LptD Thr385-Gln393 x 2 (duplication).

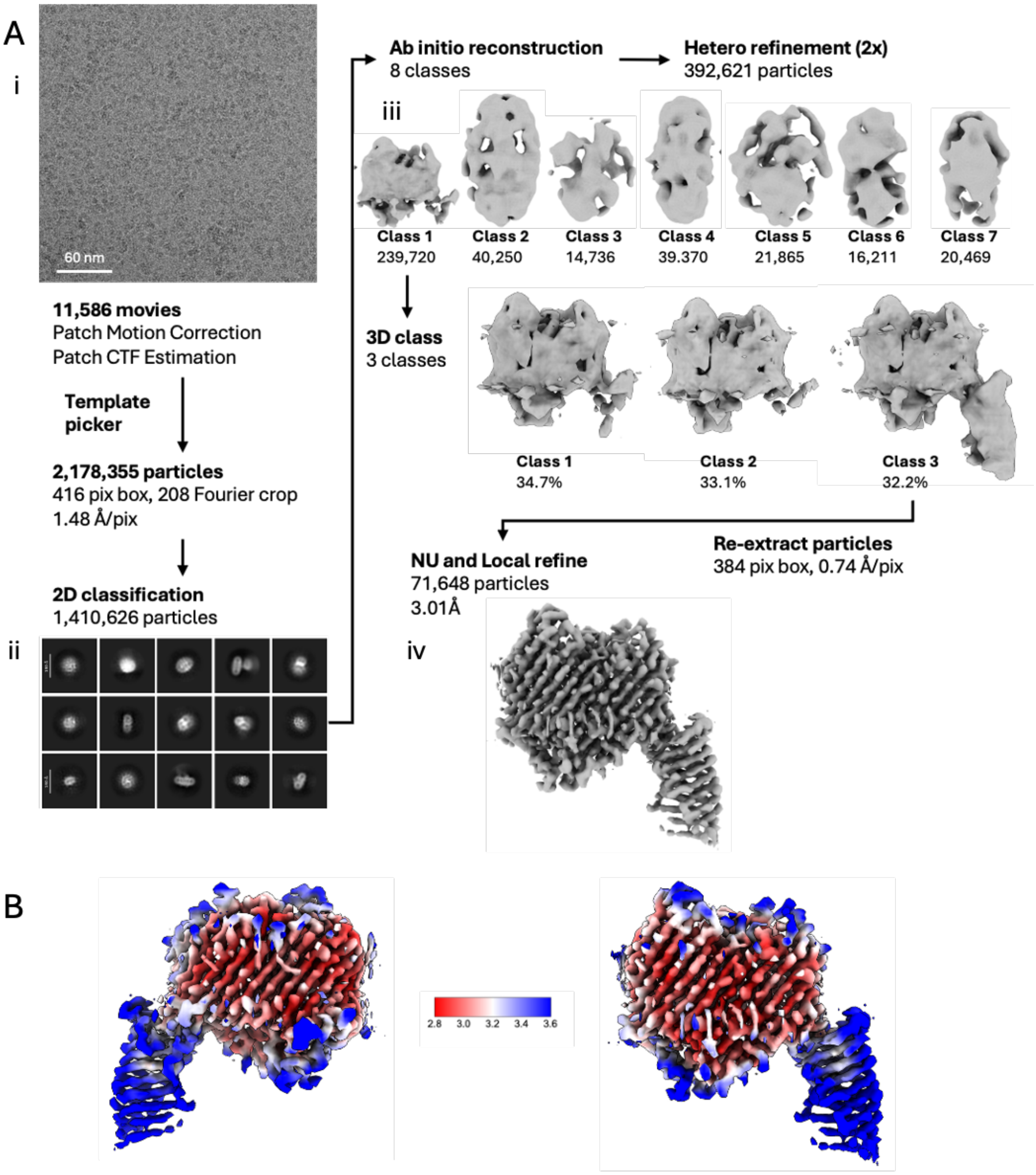

Fig. S15 Cryo-EM data processing for *Sf*LptED-*Ec*LptM-Rtp45 complex. (A) Workflow summary. 11,586 movies were collected and imported into CryoSPARC. After motion correction and CTF estimation, low-quality micrographs were excluded. Particles were extracted with Fourier cropping and subjected to two rounds of 2D classification (i, example micrograph; ii, representative 2D classes) followed by ab-initio reconstruction and heterogenous refinement using protein and junk volumes (iii, volumes). Particles were re-extracted at full resolution for non-uniform and local refinement, yielding a final 3.01Å map (iv, final volume). (B) Local resolution estimation of final map.

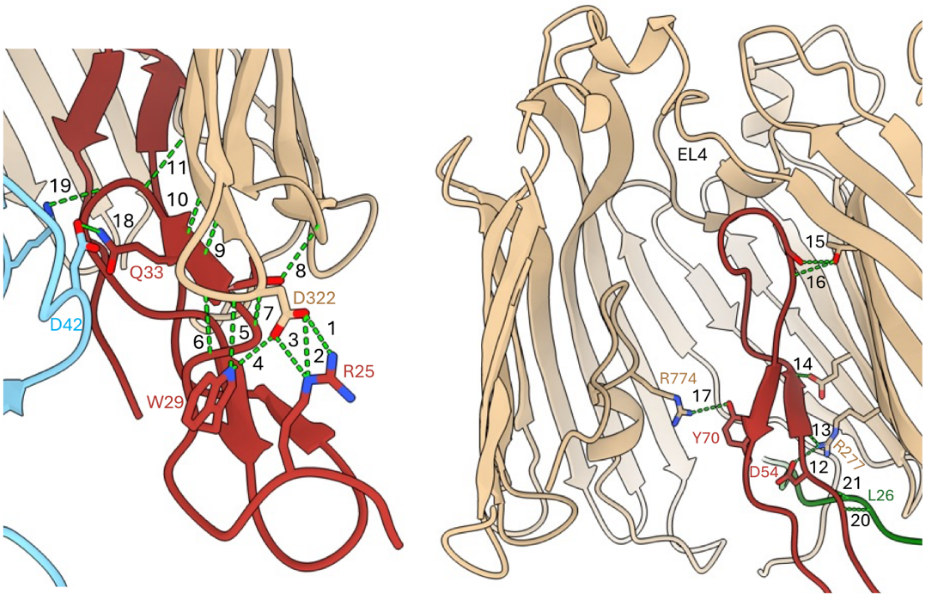

		1	2	3	4	5	6	7	8	9	10	11	12	13	14	15	16	17		18	19		20	21
RTP45	Residue	ARG	ARG	ARG	TRP	TRP	CYS	ALA	THR	GLN	GLN	SER	ASP	VAL	TYR	SER	GLY	TYR	RTP45	GLN	SER	RTP45	VAL	VAL
	No.	25	25	25	29	29	30	31	32	33	33	35	54	55	57	64	65	70		33	35		53	53
	Atom	NH2	NE	NE	NE1	NE1	O	O	OG1	N	O	N	OD1	O	N	OG	N	OH		NE2	O		O	N
LptD	Residue	ASP	ASP	ASP	ASP	ASP	GLN	ASP	GLN	VAL	VAL	SER	ARG	ARG	GLU	THR	THR	ARG	LptE	ASP	LYS	LptM	LEU	LEU
	No.	322	322	322	322	322	323	322	281	320	320	318	277	277	275	332	332	774		42	79		26	26
	Atom	OD2	OD2	OD1	OD1	O	N	N	O	O	N	O	NH1	NH1	OE1	OG1	OG1	NH2		OD2	NZ		N	O

Fig. S16 Cartoon and corresponding table showing polar interactions between Rtp45 and SfLptDE. Hydrogen bonds and salt bridges are indicated by green dashes. Colour scheme is the same as in the main text.

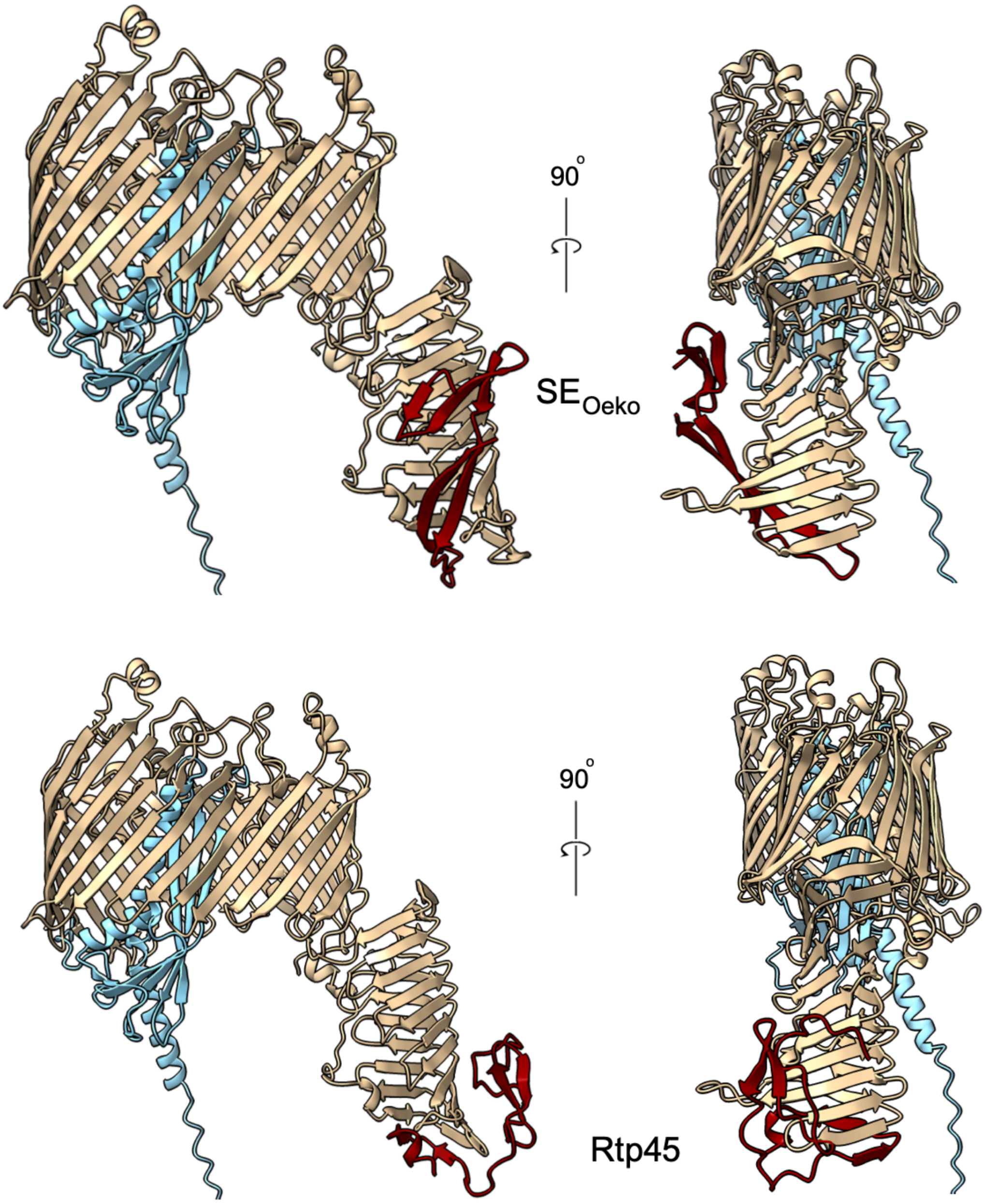

Fig. S17 AF3 models of complexes between LptDE and SE_{Oeko} (top) and Rtp45 (bottom), viewed in the OM plane. LptD is coloured tan, LptE light blue and the SE proteins maroon.

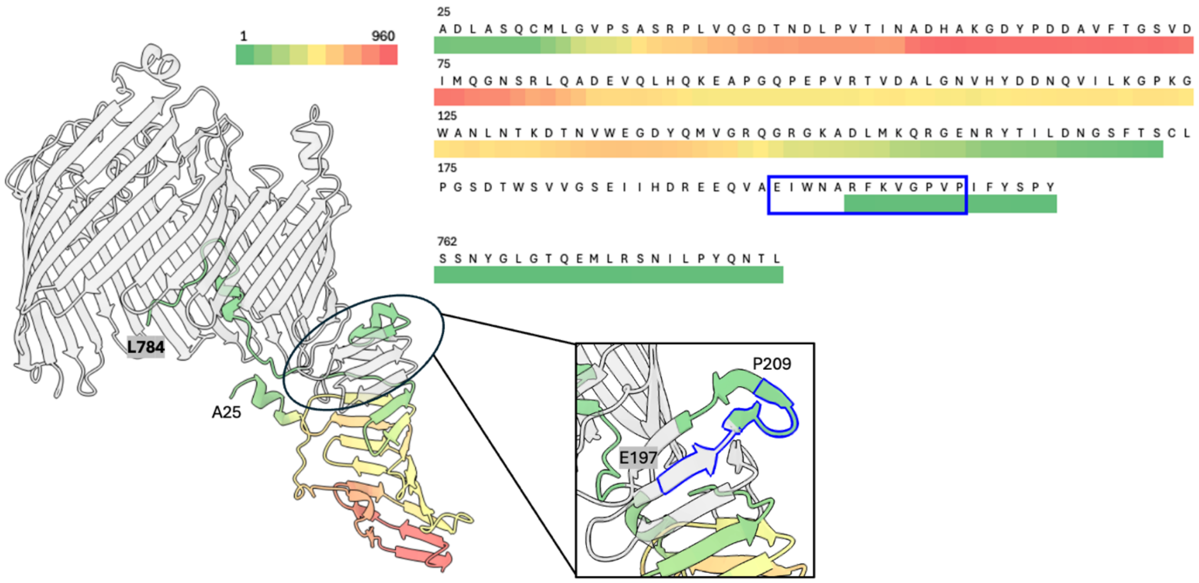

Fig. S18 Frequency analysis of LptD peptide residues detected in the LptDE-RBP sample 2 used for cryo-EM structure determination. The insert shows part of the LptD jellyroll domain in a different orientation, with the S0 segment highlighted in blue (residues Glu197-Pro209).

Table S1. Cryo-EM data collection and processing parameters.

	DE-RBP collection 1		DEM-RTP45	DEM	DE-RBP collection 2
**Data collection**					
Microscope	Titan Krios		Titan Krios	Titan Krios	Titan Krios
Detector	Falcon 4i (counting)		Falcon 4i (counting)	Falcon 4i (Selectris 10eV)	Falcon 4i (Selectris 10eV)
Voltage (kV)	300		300	300	300
Pixel size (Å)	0.74		0.74	0.74	0.74
Magnification	165,000x		165,000x	165,000x	165,000x
Total dose ($e^-/Å^2$)	35.6		50.6	50	50
Defocus range (µm)	-0.8 to -2.3		-0.8 to -2.3	-0.8 to -2.0	-0.8 to -2.0
Number of movies	10,744		11,586	7,277	6,997
**Image Processing**	**Class 1**	**Class 2**			
Initial number of particles	1,902,801	1,902,801	2,178,355	1,081,511	1,508,214
Final number of particles	134,671	55,494	71,648	112,032	158,797
Map resolution (Å)	2.81	3.20	3.01	2.78	2.76
**Refinement**	**Monomer**	**Dimer**			
MolProbity score	1.01	2.5	1.47	1.6	1.72
Clash score	2.17	5.75	2.34	2.71	5.35
Geometry					
Bad bonds (%)	0	0	0	0	0
Bad angles (%)	0	0.01	0	0	0
Stereochemistry					
Ramachandran favoured (%)	98.01	87.79	94.67	95.55	95.38
Ramachandran outliers (%)	0	0.12	0.11	0	0
Favoured rotamers (%)	91.9	84.16	91.87	89.69	71.14
Poor rotamers (%)	1.07	5.85	1.33	2.01	1.4
PDB	9RPT	9RPW	9RPS	9RPR	9RQI
EMDB	EMD-54171	EMD-54173	EMD-54170	EMD-54169	EMD-54175

Table S2 MIC values (in μg/ml) for the L- and D-variant of the S0 peptide against a panel of wild-type and *E. coli* strains with weakened OM barrier function.

Bacterial Strain[#]	MHB	
	S0 L-isomer	S0 D-isomer
E. coli lmp4213	2 – 32 (16)	16 – 64 (9)
E. coli ΔSurA	>64 (3)	>64 (3)
E. coli ΔWaaD	>64 (4)	>64 (1)
E. coli GKCW101	>64 (4)	>64 (4)
E. coli GKCW102	>64 (4)	64 - >64 (3)
E. coli ΔDsbA	>64 (1)	>64 (1)
E. coli ΔDsbC	>64 (1)	>64 (1)
E. coli ΔLptM	>64 (1)	>64 (1)
E. coli DC2	>64 (1)	>64 (1)
E. coli ATCC25922	>64 (1)	>64 (1)

[#] The panel of strains was chosen to include knockouts of non-essential genes involved in OMP biogenesis (Δ*surA* is a chaperone responsible for correct folding of the BAM complex), attenuated LPS production (Δ*waaD*) (6) and correct folding and disulfide bond formation of LptDE (Δ*dsbA*, Δ*dsbC*, Δ*lptM*) (7) GKCW102 is a hyperporinated strain with an inducible large pore (BW25113 attTn7::mini-Tn7T Kmr araC ParaBAD fhuAΔC/Δ4L) that increases OM permeability, and GKCW101 is the parent control for this (BW25113 attTn7::mini-Tn7T Kmr araC ParaBAD MCS) (8) DC2 is an antibiotic-hypersusceptible *E. coli* mutant (9). The number of experimental repeats is shown in parentheses.

Dataset S1. Peptides identified within the LptDE-RBP_{Oeko} sample. Sequences that contain part of the S0 sequence are in yellow.

Movie S1. The LptDEM structure. Map and cartoon showing bound LptM (green) within the LptD lumen.

Movie S2. LptDE with bound RBP_{Oeko}, emphasising the connection between LptD strands S0 and S1 (red). Density from dataset 2 is shown for the connecting segment.

Movie S3. LptDE with bound RBP_{Oeko}, emphasising the difference between the closed and open states of LptD. The movie starts with a density map (dataset 2) followed by a cartoon. Following the top view, RBP (magenta) is removed, and the cartoon is morphed into the closed state, with the S0 segment (red) occupying its position within the jellyroll domain. A surface

view of the closed state follows. Subsequently, the closed state morphs back into the open state and ends with a surface view (RBP not shown for clarity).

Movie S4. Rtp45 binding to LptDEM. Movie starts with a cartoon of LptDE from the LptDEM dataset (LptD in tan, LptE in blue). The barrel is clipped open and morphed into the LptDE conformation from the LptDEM-Rtp45 complex and Rtp45 (maroon) moves into position. A surface representation of RBP (magenta), from the SfLptDE-RBP_{Oeko} complex is displayed to illustrate clashes with EL4 (highlighted in green). The map from the LptDEM-Rtp45 dataset is then shown. LptM is hidden for clarity.

Supplementary references

1. A. Punjani, J. L. Rubinstein, D. J. Fleet, M. A. Brubaker, cryoSPARC: algorithms for rapid unsupervised cryo-EM structure determination. *Nat Methods* **14**, 290–296 (2017).
2. A. Punjani, H. Zhang, D. J. Fleet, Non-uniform refinement: adaptive regularization improves single-particle cryo-EM reconstruction. *Nat Methods* **17**, 1214–1221 (2020).
3. Y. Yang, *et al.*, LptM promotes oxidative maturation of the lipopolysaccharide translocon by substrate binding mimicry. *Nat Commun* **14**, 6368 (2023).
4. Q. Luo, *et al.*, Surface lipoprotein sorting by crosstalk between Lpt and Lol pathways in gram-negative bacteria. *Nat Commun* **16**, 4357 (2025).
5. M. Botte, *et al.*, Cryo-EM structures of a LptDE transporter in complex with Pro-macrobodies offer insight into lipopolysaccharide translocation. *Nat Commun* **13**, 1826 (2022).
6. T. Baba, *et al.*, Construction of *Escherichia coli* K-12 in-frame, single-gene knockout mutants: the Keio collection. *Mol Syst Biol* **2** (2006).
7. Y. Yang, *et al.*, LptM promotes oxidative maturation of the lipopolysaccharide translocon by substrate binding mimicry. *Nat Commun* **14**, 6368 (2023).
8. G. Krishnamoorthy, *et al.*, Breaking the Permeability Barrier of Escherichia coli by Controlled Hyperporination of the Outer Membrane. *Antimicrob Agents Chemother* **60**, 7372–7381 (2016).
9. D. Clark, Novel antibiotic hypersensitive mutants of *Escherichia coli* genetic mapping and chemical characterization. *FEMS Microbiol Lett* **21**, 189–195 (1984).
